# Vimentin Mediates Uptake of C3 Exoenzyme

**DOI:** 10.1371/journal.pone.0101071

**Published:** 2014-06-26

**Authors:** Astrid Rohrbeck, Anke Schröder, Sandra Hagemann, Andreas Pich, Markus Höltje, Gudrun Ahnert-Hilger, Ingo Just

**Affiliations:** 1 Institute of Toxicology, Hannover Medical School, Charité-Universitätsmedizin Berlin, Germany; 2 Center for Anatomy, Functional Cell Biology, Charité-Universitätsmedizin Berlin, Germany; CEA (Atomic and alternative energies commission), France

## Abstract

*Clostridium botulinum* C3 exoenzyme (C3) selectively inactivates RhoA/B/C GTPases by ADP-ribosylation. Based on this substrate specificity C3 is a well-established tool in cell biology. C3 is taken up by eukaryotic cells although lacking an uptake and translocation domain. Based on different approaches vimentin was identified as membranous C3-interaction partner by mass spectrometry. Vimentin in fact was partly localized at the outer surface of hippocampal HT22 cells and J744A.1 macrophages. Domain analysis identified the rod domain as binding partner of C3. Vimentin was also involved in uptake of C3 as shown by knock down of vimentin in HT22 and J774A.1 cells. The involvement of vimentin in uptake of C3 was further supported by the findings that the vimentin disruptor acrylamide blocked uptake of C3. Vimentin is not only a major organizing element of the intermediate filament network but is also involved in both binding and uptake of C3 exoenzyme.

## Introduction

Exoenzyme C3 from *Clostridium botulinum* is the prototype of Rho-ADP-ribosylating transferases produced by different bacterial strains such as *Clostridia, Bacilli* and *Staphylococci* [1], [2], [3]. The transferred ADP-ribose moiety at acceptor amino acid Asn-41 renders Rho functionally inactive and halts Rho-dependent down-stream signaling [4], [5]. The most prominent consequence is the reorganization of the actin cytoskeleton accompanied by morphological changes [6], [7]. ADP-ribosylation is highly specific as preferentially RhoA, B and C from the Ras superfamily of small GTPases are modified. This specificity is the basis for its use as cell biological tool to inactivate RhoA/B/C function in intact cells, still in the time of knock down techniques [8], [9]. In addition to its enzymatical activity, C3 exhibits an enzyme-independent biological activity to stimulate axonal and dendritic growth as well as branching in primary hippocampal neurons [10].

Cellular uptake of bacterial protein toxins such as diphtheria toxin or cholera toxin takes place via receptor-mediated endocytosis [11]. Cell entry is mediated by specific functional domains like receptor binding, transport and translocation domain. After endocytosis release into the cytoplasm is mediated by translocation through a pore formed in an acidified vesicle (short trip uptake) or through retrograde transport followed by release from the endoplasmic reticulum (long trip) [11]. Although C3 is referred as toxin it clearly lacks the canonical domains of bacterial protein toxins, which are in charge of cellular uptake. The crystal structure of C3 does not give any hints how binding to cells and uptake is mediated [12]. Nevertheless, C3 in fact reaches the cytoplasm of intact cells as intracellular Rho is ADP-ribosylated. The cellular receptor as first step of C3 cell entry and the exact endocytosis process are unknown. Recently, it was shown that the uptake of C3 in macrophage-like cells could be inhibited by bafilomycin an inhibitor of the vacuolar ATPase, indicating the involvement of an acidic compartment during translocation [13].

Sensitivity of various cell lines towards C3 differs dramatically. Low concentrations of C3 are sufficient to selectively intoxicate cultured macrophage-like murine J774A.1 cells [14] and human promyelotic leukaemia (HL-60) cells [13]. The most sensitive cells seem to be primary cells like neurons [10], astrocytes and microglia [15].

The interaction of a bacterial protein toxin with its cellular receptor is the key step for cell entry. The nature of toxin receptors is very broad, covering numerous lipids or lipid derivatives or membranous proteins or glycoproteins [16]. Many years ago it was demonstrated that post-translational modification of the receptor is important for toxin-receptor binding [17], [18]. For example the monosialoganglioside G_M1_ is the ganglioside that strongly interacts with cholera toxin [19], [20]. In this study, the hippocampal-derived cell line HT22 was used. Protein overlay assays, binding and uptake assays combined with siRNA knock down as well as mass spectrometrical analyzes established vimentin as C3 binding partner at the cell surface.

## Materials and Methods

### Cell Culture

Murine hippocampal HT22 cells, which were a generous gift from Prof. Dr. Carsten Clumsee (Institute for Pharmacology and Toxicology, Philipps University Marburg, Germany) [21] were cultivated in Dulbecco’s modified essential medium (Biochrom, +10% FCS, 1% penicillin, 1% streptomycin, and 1 mM sodium pyruvate). J774A.1 mouse macrophages (purchased from American Type Culture Collection ATCC: TIB-67) were cultivated in RPMI 1640 Medium (Biochrom; with 10% FCS, 1% penicillin, 1% streptomycin, and 1 mM sodium pyruvate). Cells were maintained at 37°C and 5% CO_2_. Upon confluence, cells were passaged. Primary astrocytes were prepared and cultivated as described previously [22].

Pronase was from Roche (Roche Applied Science, Mannheim, Germany). For pronase treatment cells 300,000 HT22 cells were seeded onto 3.5 cm plates and grown for 24 h at 37°C and 5% CO2. The medium was removed and cells were washed with PBS. Cells were cultivated in DMEM with 1% FCS and preincubated with 500 µg/ml of pronase for 30 min at 4°C. Serum (5% FCS) was added to detached cells to block pronase activity and cells were then washed with PBS and incubated with C3 (500 nM) for 1 h at 4°C.

### Western Blot Analysis

For Western blot analysis the following primary antibodies were used: RhoA was identified using a mouse monoclonal IgG from Santa Cruz Biotechnologies (Santa Cruz, USA). Identification of C3 was achieved by a rabbit polyclonal antibody (affinity purified), which was raised against the full length toxin C3bot (accession no. CAA41767). Vimentin was identified using rabbit monoclonal anti-vimentin antibody (Epitomics, Carlifornia, USA). A polyclonal antiserum against GFAP used to label astrocytes and Actin (used as loading control) was purchased from Sigma (Sigma-Aldrich Chemie GmbH, Munich, Germany). Western blot analyses were performed as described previously [23].

### Blot Overlay Assay

HT22 cells were washed and scraped into Triton X-100 buffer. The obtained suspension was shaken at 37°C for 10 min. Ultrasonic disruption was performed in a cycle of 10×5 sec, 5×10% sonic energy using a sonotrode (Bandelin Electronic, Berlin, Germany). Lysates were centrifuged at 2000 g for 10 min at 4°C. The particulate fraction was resuspended in 50 µl Triton X-100 buffer [150 mM NaCl, 50 mM Tris and 1% Triton-X 100] and samples were separated by 10% SDS-PAGE and transferred to nitrocellulose membranes. After incubation with blocking buffer [5% powdered milk, in Tris-Buffered Saline with Tween (TBST)], membranes were probed with 10 µg/ml purified C3 in blocking buffer overnight at 4°C. After extensive washing with TBST, membranes were incubated with anti-C3 antibody, followed by HRP-conjugated secondary antibody, and detected by ECL. As a positive control, whole cell lysates with 10–20 ng C3 was loaded in the same nitrocellulose membranes and immunoblotted with C3 antibody. For the chemiluminescence reaction, ECL Femto (Pierce, Thermo Fisher Scientific Inc., Rockford, IL, USA) or Immobilon (Millipore, Schwalbach, Germany) was used.

### 2D-Gel Mass Spectrometry Analysis

HT22 cells were washed and scraped into Triton X-100 buffer. The obtained suspension was shaken at 37°C for 10 min. Ultrasonic disruption was performed in a cycle of 10×5 sec, 5×10% sonic energy using a sonotrode (Bandelin Electronic, Berlin, Germany). Lysates were centrifuged at 2000 g for 10 min at 4°C. The pellets were resuspended in 50 µl Triton X-100 buffer and separated by Nonequilibrium pH gradient electrophoresis (NEPHGE)/SDS-PAGE. NEPHGE/SDS-PAGE and protein blotting was carried out by WITA protolays (WITA GmbH, Germany). 2D-gels were subjected to either protein electro transfer onto PVDF membrane, or colloidal coomassie brilliant blue (CBB-G250) staining. The PVDF membrane was used for a C3 overlay assay and the colloidal CBB stained gel for mass spectrometry analysis. For alkylation of cysteine residue, dehydrated gel pieces were incubated with 20 µl 15 mM DTT (Sigma-Aldrich Chemie GmbH, Munich, Germany) for 45 min at 56°C. After dehydration with acetonitrile, gel pieces were incubated with 100 mM iodoacetamide (Sigma-Aldrich Chemie GmbH, Munich, Germany) for 15 min at room temperature in the dark. Subsequently, gel pieces were extracted with 200 µl 50% (v/v) acetonitrile in 0.2% (v/v) TFA for 30 min at 400 rpm followed by dehydration with 100 µl of 100% acetonitrile. Peptide containing supernatants were pooled and dried using vacuum evaporation.

### HT22 cell lysate overlay

HT22 cells were washed and scraped into Triton X-100 buffer [150 mM NaCl, 50 mM Tris and 1% Triton-X 100]. The obtained suspension was shaken at 37°C for 10 min. Ultrasonic disruption was performed in a cycle of 10×5 sec, 5×10% sonic energy using a sonotrode (Bandelin Electronic, Berlin Germany).

50 µg of recombinant C3 protein were separated by native 10% PAGE and transferred to nitrocellulose membranes. After Western blot the transfer efficiency was checked with ponceau staining and as a negative control the membrane was cut below the C3 protein band. After incubation with blocking buffer [5% powdered milk, in Tris-Buffered Saline with Tween (TBST)], membranes were probed with HT22 whole cell lysate for 2 h at 4°C. After extensive washing with TBST, membranes were incubated with 20 mM NH_4_HCO_3_, 10% (v/v) acetonitrile and digestion by trypsin gold (Promega GmbH, Mannheim, Germany) was continued over night at 37°C. The reaction was stopped with 50 µl 50% (v/v) acetonitrile in 0.5% (v/v) TFA for 10 min and shaking at 400 rpm. Peptide containing supernatants were dried using vacuum evaporation.

### MALDI-ToF/ToF analysis

Peptides of 1D gels, 2D gel spots and the HT22 cell lysate probed membranes were analyzed using MALDI-MS/MS as described previously [24]. Internal calibration on autolytic porcine trypsin peptides was applied for precursor MS spectra and external calibration with Glu-Fib fragments was used for MS/MS spectra. Data analysis was performed with Protein Pilot 3.0 and proteins were identified using Mascot search algorithm and the Swissprot database (taxonomy *Mus musculus*) with a maximum of two missed cleavages and with carbamidomethylation of cysteins as static and oxidation of methionine as variable modification. Peptide mass tolerance was set to ±20 ppm and fragment mass tolerance to ±0.7 Da.

### LC-MS analysis

Peptides of 1D gel digestion were analyzed using LC-MS analysis. Therefore, dried samples were resuspended in 50 µl 2% (v/v) ACN, 0.1% (v/v) TFA. After centrifugation for 2 min at 13 500 g, the supernatant was transferred to an LC sample vial and an appropriate amount of each sample was injected into the LC system (Ultimate 3000 RSLC system, Dionex). Peptides were loaded on a trap column (C18 material, 2 cm length, 75 µm ID, Acclaim PepMap, Dionex) with 6 µl/min and washed with 0.1% (v/v) TFA loading buffer. After 5 min the trap column was switched in line with the nano flow separation column (C18 material, 50 cm length, 75 µm ID, Acclaim PepMap, Dionex) and peptides were eluted with a flow of 250 nl/min and a linear gradient of elution buffer A (0.1% (v/v) formic acid) and elution buffer B (80% (v/v) acetonitrile, 0.1% (v/v) formic acid) from 4–50% elution buffer B in 65 min, from 50–90% elution buffer B in 5 min. Then the column was flushed isocratically with 90% elution buffer B for 10 min, 90–4% elution buffer B in 5 min and finally equilibrated for 15 min in 4% elution buffer B. The LC system was online connected to the nanoESI source of an LTQ Orbitrap velos (Thermo Fisher Scientific, USA). MS precursor scans were acquired from 300–1700 m/z in profile mode with a resolution of 60,000 at 400 m/z in the orbitrap mass analyzer. The top five most intense ions of charge state ≥2 and a minimum signal threshold of 1,800 counts were selected for HCD fragmentation with normalized collision energy of 38%. Fragments were scanned out in the orbitrap mass analyzer in centroid mode with a resolution of 7,500 at 400 m/z. Data were analyzed with Proteome Discoverer 1.2 (Thermo Fisher Scientific, USA). Spectra were searched with Mascot search algorithm and the Swissprot database with maximum of two missed cleavage sites, propionamide of cysteine as static and oxidation of methionine, acetylation of lysine and phosphorylation of serine and threonine as fixed modification. Peptide mass tolerance was set to ±5 ppm and fragment mass tolerance to 0.8 Da.

### Expression and purification of recombinant C3 protein

C3 wild type and *Clostridium botulinum* derived mutant C3-E174Q (carrying a point mutation from glutamate to glutamine at AA 174) were expressed as recombinant GST-fusion proteins in *E. coli* TG1 harbouring the respective DNA fragment (gene of *Clostridium botulinum* C3, accession no. X59039) in the plasmid pGEX-2T (GE Healthcare Europe GmbH, Freiburg, Germany) as described previously [22]. ADP-ribosyltransferase activity was measured by an *in vitro* ADP-ribosylation assay.

### C3 binding assay

For the binding assays cultivated cells were seeded onto 3.5 cm plates at a concentration of 300,000 cells/ml and grown for 24 h at 37°C and 5% CO2. The medium was removed and cells were washed with PBS. 300,000 cells/ml were exposed to 100 or 500 nM of C3 for 1 h at 4°C. Subsequently, cells were stringent washed three times with PBS. Cells were scraped into Laemmli sample buffer. The obtained suspension was shaken at 37°C for 10 min. Ultrasonic disruption was performed in a cycle of 10×5 sec, 5×10% sonic energy using a sonotrode (Bandelin Electronic, Berlin, Germany). The lysate was then incubated at 95°C for 10 min and submitted to SDS-PAGE and Western blot analysis against α-C3 and β-actin.

### Expression and purification of recombinant mouse vimentin proteins

Plasmids of mouse vimentins provided by Prof. Dr. Yi-Ling Li, Institute of Biomedical Sciences, Genomics Research Center, Academia Sinica, Taipei, Taiwan were used [25]. The plasmids were transformed into BL21 (DE3) cells. Induction was with 1 mM IPTG at 37°C for 3 h. Recombinant vimentin proteins were purified as described by Machery-Nagel for His-tag protein purification (Ni-IDA 2000 Packed Columns Protino, Machery-Nagel GmbH & Co. KG, Düren, Germany). Eluted proteins were analyzed by 15% SDS-PAGE, stained with Coomassie blue and submitted to Western blot analysis against α-penta-His (Qiagen (catalog no. 34660), Hilden, Germany).

### Immunoprecipitation of C3-vimentin complex

Purified recombinant vimentin (2 µg/ml) and C3 exoenzyme (2 µg/ml) were incubated in 1 ml immunoprecipitation buffer (20 mM Tris HCl pH 7.2, 50 mM NaCl, 3 mM MgCl, 1% NP40, 100 µM PMSF, 1% protease inhibitors) for 2 h at 4°C under rotation. Immunoprecipitation of C3-vimentin complex were done with C3 antibody followed by incubation with 50 µl protein A/G PLUS-agarose beads (Santa Cruz Biotechnology, Inc.) for 45 minutes. Agarose beads were spun down at 10,000 g for 5 min, washed 2 times with immunoprecipitation buffer and re-suspended in SDS-PAGE sample buffer. Beads proteins and supernatant from the last wash step were separated by 10% SDS-PAGE followed by Western blot analysis with anti-C3 and anit-vimentin (Epitomics, Carlifornia, USA).

### Vimentin binding assay

An equal amount of purified vimentin head (amino acids 1 to 101), rod (amino acids 102 to 410), and tail (amino acids 411 to 466) domains in the His-tagged forms were separated by 15% SDS-PAGE and transferred to nitrocellulose membranes. After incubation with blocking buffer [5% powdered milk, in TBST], membranes were probed with 10 µg/ml purified C3 in blocking buffer overnight at 4°C. After extensive washing with TBST, membranes were incubated with anti-C3 antibody, followed by HRP-conjugated secondary antibody, and detected by ECL. For the chemiluminescence reaction, ECL Femto (Pierce, Thermo Fisher Scientific Inc., Rockford, IL, USA) or Immobilon (Millipore, Schwalbach, Germany) was used.

### Cell surface biotinylation of proteins

The surface membrane biotinylation of cells surface proteins and isolation was performed using the Cell Surface Protein Isolation Kit (Pierce, Rockford, IL) as directed by the manufacturer. Cultured HT22 cells grown in 75 cm2 culture flasks. Conditioned medium was removed; cells were washed with 2×2 ml of PBS, and surface proteins were labeled with a non-cell-permeable sulfo-NHS biotin analog (500 µl at 500 µg/ml PBS, Pierce) under gentle shaking at 4°C for 1 h. After washing, cells were incubated with 500 µl quenching solution. Washed cells were lysed with 500 µl of lysis buffer, collected with a cell scraper, and clarified by centrifugation (10,000×g, 2 min, 4°C). To precipitate biotin-labeled cell surface proteins, lysate was added to immobilized NeutrAvidin gel and incubate for 1 h under gentle shaking. Complexes were analyzed by 10% SDS-PAGE and Western blot analysis. Further biotinylated samples were resolved by SDS-PAGE. The gel was stained with Coomassie blue (Bio-Rad Laboratories GmbH, Munich, Germany) and bands at 55 kDa were excised and analyzed by mass spectrometry.

### Cell surface expression of vimentin using Fluorescence-activated cell sorting (FACS)

Cells (2×10^5^ cells/ml DMEM) were incubated at 37°C, 5% CO2 for 24 h. The medium was removed and cells were washed with PBS and incubated with accutase (1 ml per 3.5 cm plates) (Sigma-Aldrich Chemie GmbH, Munich, Germany) for 3 min at 37°C. Then accutase activity was blocked with ice cold PBS. Detached cells were centrifuged and cell pellet was resuspended in PBS with 5% FCS and incubated for 15 min on ice. Cells were washed in PBS and incubated for 60 min on ice with rabbit monoclonal anti-vimentin antibody (Epitomics, Carlifornia, USA). Oregon green-488 conjugated goat anti-rabbit antibody alone was used as negative control. Cells were washed in PBS and incubated for 30 min on ice with Oregon Green 488-conjugated goat anti-rabbit antibody (Molecular Probes, Life Technologies GmbH, Darmstadt, Germany). Cell surface expression was analyzed using a FACScan (FACScan flow cytometer, Becton Dickinson). Ten thousand events were monitored per condition.

### Cell surface binding of C3-A1C/E174Q/K211C-FITC using Fluorescence-activated cell sorting (FACS)

For testing the interaction of C3 with HT22 and J774A.1 cells using FACS cytometry, recombinant C3-A1C/E174Q/K211C protein was labeled with 5-flourescein isothiocyanate (FITC) microscale protein labeling kit (AnaSpec Inc, Fremont, Ca, USA) according to the manufacturer’s instructions. For the flow cytometry analysis we used a recombinant enzyme deficient C3-A1C/E174Q/K211C with two free amines to conjugate fluorescein- 5-isothiocyanate to C3bot. C3 deficient enzyme was chosen to avoid morphological changes even after prolonged incubation time.

Cells (2×10^5^ cells/ml DMEM) were incubated at 37°C, 5% CO2 for 24 h. Cells were harvested in cold PBS and blocked with PBS containing 5% FCS for 15 min on ice. Cells were washed in PBS and incubated for 60 min on ice with 500 nM C3-E174Q-FITC. Cells were washed three times with PBS and the C3 binding was analyzed using a FACScan (FACScan flow cytometer, Becton Dickinson). Ten thousand events were monitored per condition.

### siRNA transfection

HT22 and J774A.1 cells were plated at 1.5×10^5^ onto 3.5 cm plates and grown for 24 h at 37°C and 5% CO2. The medium was removed and cells were washed with PBS. Cells were transfected with Vim siRNA (On-TARGETplus Smart pool, Dharmacon, USA) at a final concentration of 100 or 200 nM with jetPRIME siRNA transfection reagent (Peqlab Biotechnologie GmbH, Erlangen, Germany) according to the manufacturer’s instructions. A scrambled siRNA was used as a control. Scrambled siRNAs contained a random sequence content and with no calculated target gene specificity.

For pulse chase experiment with C3, HT22 cells were 48 h post-transfectional incubated with 500 nM C3 for 60 min by 4°C. Afterwards C3 containing medium was removed. Cells were washed three times with PBS and fresh medium was added. Cells were cultivated for 48 h and then harvested.

For RhoA gel-shift assay, J774A.1 cells were 48 h post-transfectional incubated with 500 nM C3 for 4 h at 37°C. After this incubation, cells were washed with PBS and scraped into Laemmli sample buffer.

### Immunocytochemistry

HT22 and J774A.1 cells seeded on cover slips were washed with PBS and subsequently fixed in 4% formaldehyde in phosphate buffered saline (PBS) (pH 7.4) at room temperature for 20 min. Cells were then washed and permeabilized with 0.3% (w/v) Triton X-100 in PBS supplemented with 5% BSA. Vimentin was stained by α-vimentin antibody and Oregon green 488 conjugated secondary antibody for 1 h at room temperature either. F-actin staining was performed using rhodamine-conjugated phalloidin (30 µg/ml) for 30 min at room temperature. Then, a 0.1 µg/ml solution of DAPI in phosphate-buffered saline supplemented with 0.1% (w/v) Tween-20 was used for nuclei staining for 30 min at 37°C. Cells were analyzed by fluorescence microscopy using a Zeiss Axiovert 200 M and Leica TCS 2 inverted confocal microscope.

In the case of co-localization of C3-FITC and vimentin, cells were treated with 1000 nM C3-FITC for 2 h in serum-free DMEM and washed with PBS, then fixed with 4% PFA for 20 min at room temperature. Cells were then washed and permeabilized with 0.02% saponin in PBS supplemented with 5% BSA. Vimentin was stained by α-vimentin antibody and Alexa-555 conjugated secondary antibody for 1 h at room temperature either. DAPI was used for nuclei staining. Cells were analyzed using a Leica TCS 2 inverted confocal microscope.

Primary astrocytes were fixed and stained as described previously [22].

### Confocal laser scanning microscopy

For image acquisition a Leica TCS SL confocal laser scanning microscope using a 63× oil immersion objective was used. Fluorescent dyes were excited at a wavelength of 488 nm (green fluorescence), 543 nm (red fluorescence), and 405 nm (blue fluorescence) respectively. Images were captured at a resolution of 1024×1024 pixels.

### Reproducibility of the experiments and statistics

All experiments were performed independently at least three times. Results from representative experiments are shown in the figures. Values (n ≥3) are means ± SEM. The two-sided unpaired Student's *t* test was used throughout the study to assess the statistical significance of the differences between two sets of data. Differences were considered to be statistically significant at p≤0.05 (* = p≤0.05 and ** = p≤0.01).

## Results

### C3 binds to proteinaceous structures at intact HT22 cells

**Figure 1:**
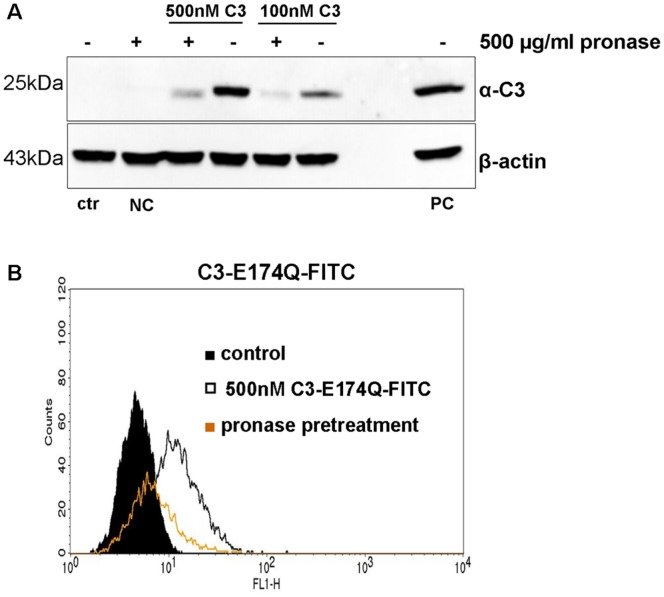
Binding of C3 to HT22 cells after pronase treatment. A) Pronase pre-incubated HT22 cells were exposed to 100 or 500 nM of C3 for 1 h at 4°C. Subsequently, β-actin and bound C3 were detected by Western blot. NC = negative control without C3, PC = positive control lysate with 10 ng C3. One representative experiment is shown (n = 3 independent experiments). B) Pronase-treated HT22 cells were exposed to 500 nM of C3-E174Q-FITC for 1 h at 4°C and bound C3- E174Q-FITC was analyzed by FACS.

To check whether C3 binding to intact HT22 cells was dependent on proteinaceous cell surface structures or not, cells were treated with pronase prior to binding to C3. Detached cells were washed and pronase activity was inhibited by addition of serum (Fig. S1). Binding of C3 to HT22 cells was detected by Western blot using C3-specific antibody (Fig. 1A) and flow cytometry by fluorescently labeled C3 (Fig. 1B). Enzyme deficient C3 (C3-E174Q) was chosen in the flow cytometry assay to avoid morphological changes. Binding of C3-E174Q to HT22 cells was similar to the binding of C3 (Fig. S2). Both approaches Western blot and flow cytometry showed strong decrease in C3 binding suggesting proteinaceous structures as membrane binding partner of C3.

### Analysis of C3-binding proteins

**Figure 2:**
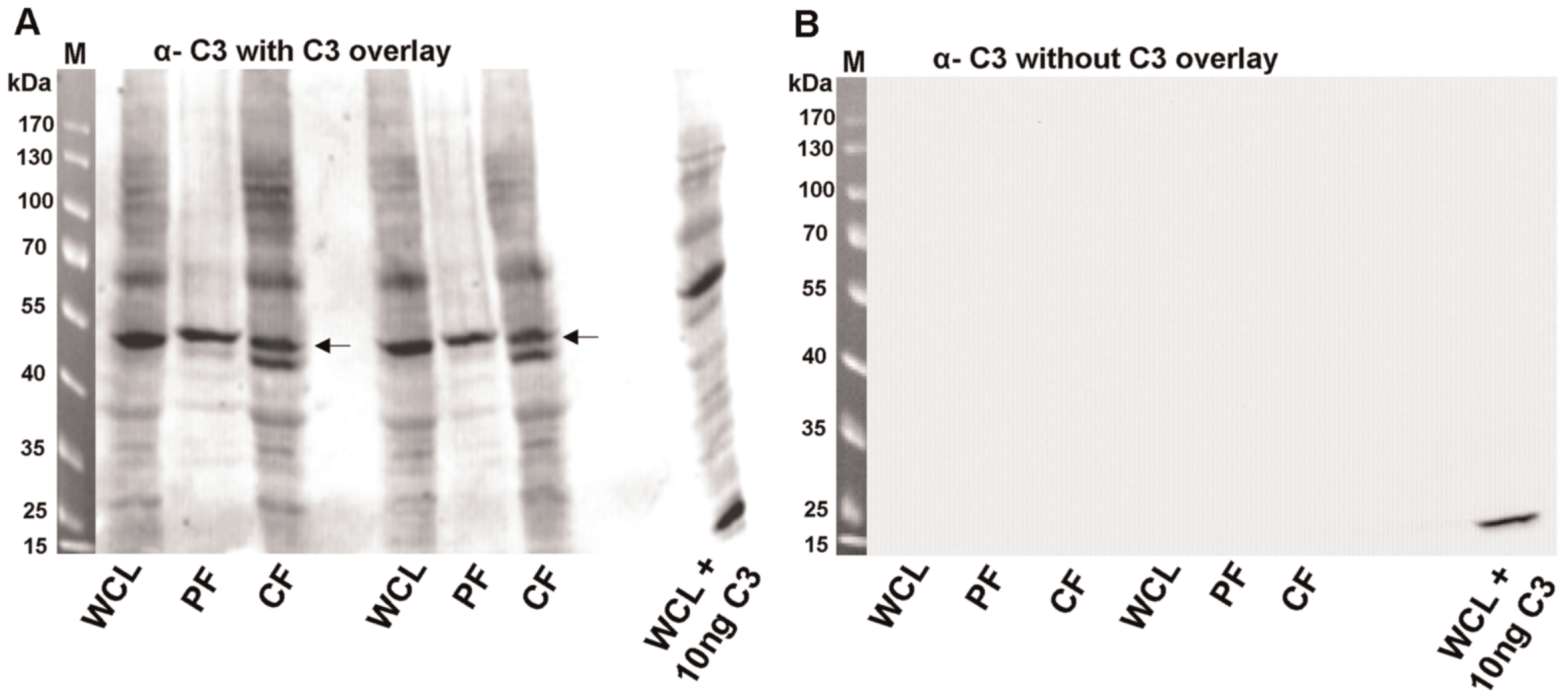
C3-overlay (binding of C3 to HT22 proteins). A) Whole cell lysate, cytosolic fraction or particulate fraction from HT22 cells were generated as described in material and methods followed by separation through SDS-PAGE and transfer onto nitrocellulose. Nitrocellulose was incubated with 10 µg/ml of C3 for 60 min at 4°C. After washing bound C3 was detected by anti-C3. Arrows indicate the protein of interest (55 kDa). B) The right panel shows the anti-C3 Western blot without C3-overlay. M = molecular mass marker, WCL = whole cell lysate, PF = particulate fraction, CF = cytosolic fraction, WCL +10 ng C3 = C3 was added to whole cells lysate prior to SDS-PAGE and blotting to generate a positive C3 signal.

To identify the putative surface receptor, cell lysates and the particulate cell fractions from HT22 cells were separated by SDS-PAGE followed by electroblotting onto nitrocellulose. The nitrocellulose was then overlaid with C3, washed and bound C3 was detected by anti-C3. Bound C3 was detected at a molecular mass range of about 110 kDa and of 45–55 kDa (cell lysates), at about 55 kDa as one distinct twin band (particulate fraction) and at about 55 and 110 kDa (cytosolic fraction) (Fig. 2A, left panel). Thus, binding of C3 to immobilized proteins appeared to be specific since no signal was identified in the control sample without C3 (Fig. 2, right panel).

To dissolve the 55 kDa bands, the particulate fraction of HT22 cells was separated by two-dimensional gel electrophoresis (2DE), transferred to PVDF membrane and overlaid with C3 followed by probing with anti-C3. Seven spots were detected in the overlay-blot (Figure S3). To identify these spots, the corresponding gel areas were cut out from the 2D gel, digested with trypsin and subjected to LC-MS identification. The spots were identified as: heterogeneous nuclear ribonucleoprotein A3 (HRPC3), heat shock cognate 71 kDa protein (HSP7C), vimentin, heterogeneous nuclear ribonucleoprotein F (HNRPF), β-actin and nucleophosmin (Fig. S3). A complete list of all identified proteins is available as Table S1.

To confine the putative C3 binding partners a reverse experiment was set up. C3 separated by non-denaturating SDS-PAGE was blotted onto nitrocellulose membrane and overlaid with HT22 lysates. After intensive washing proteins bound to the immobilized C3 (25 kDa band) were digested and identified by LC-MS. The proteins identified were: histone H2B type 1F (H2B1F), 40S ribosomal protein S1 (RS19), heterogeneous nuclear ribonucleoproteins A2/B1 (HNRNPA2B1), β-actin and vimentin. Notably, β-actin and vimentin were also identified in the 2D gel experiment. A complete list of identified proteins including accession No, Mascot identification score, number of identified peptides, mass, isoelectric point and sequence coverage is available as Table S2. In further experiments we focused on vimentin because of the high validity of identification and due to the reported presence of vimentin at the cell surface [26]–[30]. In addition, in the C3-overlay assay we observed a positive signal at 55 kDa. Vimentin (55 kDa) does match to this molecular weight instead of actin (43 kDa). Further, it is known that vimentin is involved in both endocytosis and vesicular trafficking and plays a role in uptake of pathogens [31]–[33]. Therefore, it is conceivable that vimentin might be involved in C3 binding and internalization.

### C3 interacts with the intermediate filament protein vimentin

**Figure 3:**
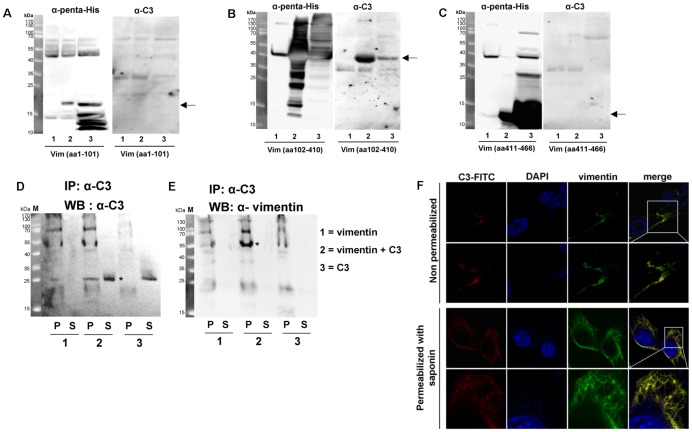
Binding of C3 to the rod domain of vimentin. His-tagged vimentin domains (A) head: amino acids 1 to 101; (B) rod: amino acids 102 to 410; (C) tail: amino acids 411 to 466 were expressed in *E. coli*. Crude extracts from non-induced *E. coli* (lane 1), crude extracts from induced *E. coli* (lane 2) and His-tagged vimentin domains purified through Ni-IDA column (lane 3) were separated by 15% SDS-PAGE and electroblotted. Left panels of A, B and C show probing with penta-His polyclonal antibody to detect vimentin domains. Right panels of A, B and C show probing with anti-C3 to detect bound C3. Predicted apparent mol masses of each vimentin domain are indicated by arrow. Arrows indicate the protein of interest. D) Immunoprecipitation of C3 with C3 antibody. Full length recombinant vimentin was incubated with C3 in solution. The vimentin-C3 complex was immunoprecipitated with C3 antibody. P = pellet of agarose beads, S = supernatant. E) Immunoprecipitation of vimentin with C3 antibody and detection of vimentin. P = pellet of agarose beads, S = supernatant. F) For imaging vimentin and C3 at the cell surface (upper panel), cells were incubated with C3-FITC for 1 h at 4°C. After washing with PBS cells were fixed (without permeabilization) and incubated with vimentin antibody following by an Alexa-555 conjugated secondary antibody. For imaging intracellular vimentin (lower panel), cells were fixed, permeabilized and incubated as described above. For imaging intracellular C3, living cells were incubated with C3-FITC for 1 h at 37°C. After washing with PBS cells were fixed, permeabilized with 0.02% saponin for 30 min and imaged by confocal microscopy.

To confirm the interaction between C3 and vimentin, additional blot overlay assays with recombinant vimentin were performed. Using the *E. coli* expression system recombinant His-tagged mouse vimentin and vimentin fragments (rod domain (aa102–410), head (aa1–101) and tail domain (aa411–466) were generated. Expression was checked with anti-His-tag (Fig. 3A–C *).* Comparable amounts of recombinant vimentin fragments were electrophoretically separated, blotted and then overlaid with C3 followed by probing with anti-C3. C3 only bound to the rod domain (Fig. 3B) but neither to head nor to tail domains of vimentin (Fig. 3A and C). To confirm the interaction between C3 and vimentin, immunoprecipitation assay with antibodies as indicated were performed (Fig. 3D and E). Results showed that C3 and vimentin form a protein complex, since the immunoprecipitation of C3 resulted in a positive signal at 25 kDa (C3 exoenzyme, asterisk, Fig. 3D) and enriched vimentin protein at 55 kDa (asterisk, Fig. 3E). To confirm the interaction between C3 and vimentin we additionally performed co-localization studies. The colocalization of vimentin and C3 at the cell surface was compared using confocal microscopy. At the cell surface only a weak signal for vimentin and C3-FITC was observed. It seems that the co-localization takes place at the focal adhesion sites (Fig. 3F, upper panel). Nevertheless, the intracellular localization of C3-FITC and vimentin was compared. Vimentin protein was visualized with an anti-vimentin antibody, whereas C3 were visualized using the conjugated FITC. A diffuse punctuate staining was observed indicating that both the C3-FITC and vimentin co-localized (Fig. 3 F, lower panel).

**Figure 4:**
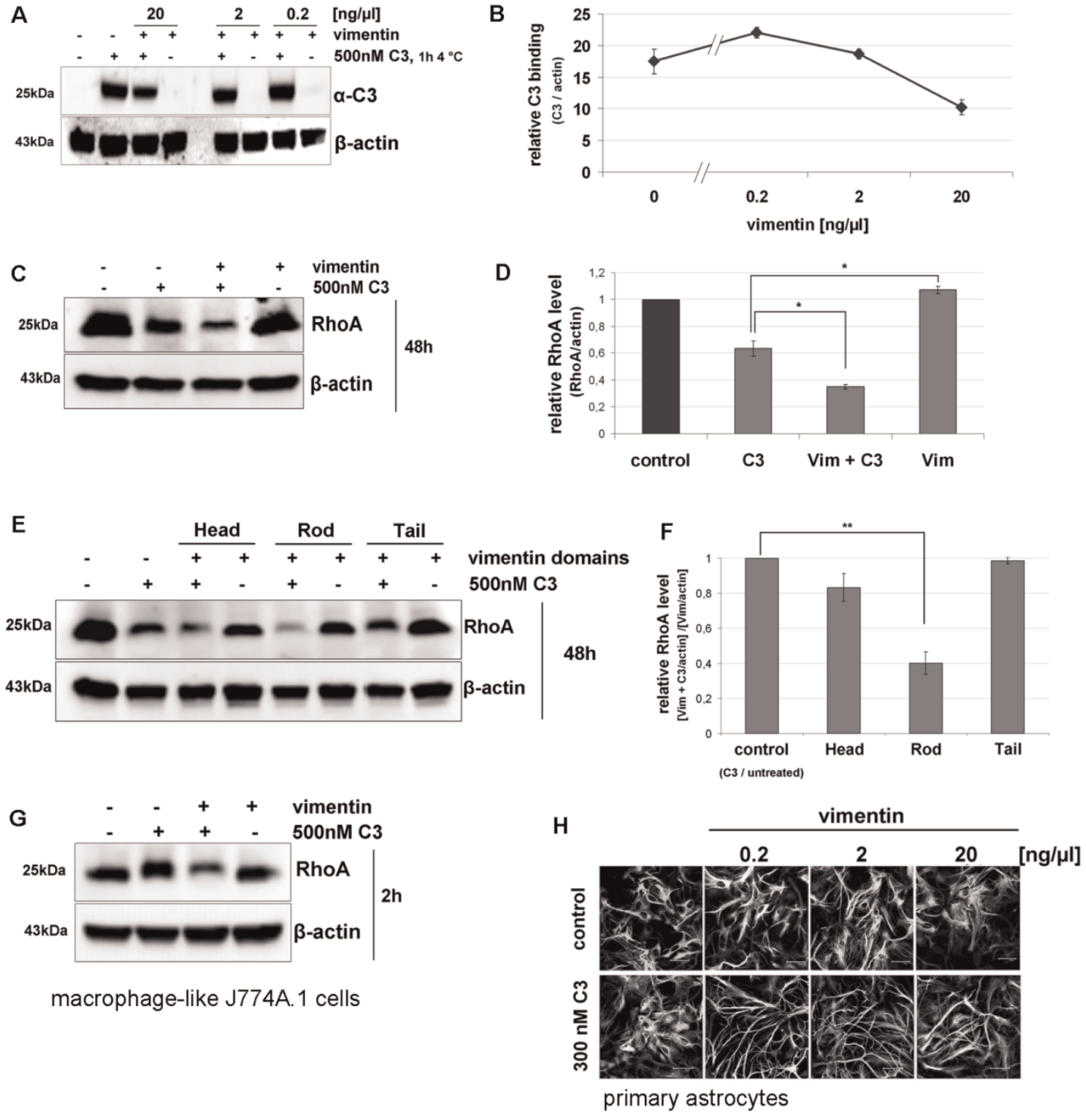
Binding and uptake of C3 in cultivated cells dependent on vimentin. A) The Western blot shows the binding of C3 in presence and absence of extracellular added vimentin (n = 3 independent experiments). B) Densitometric evaluation of C3 (from A) and adjustment to the corresponding actin band are shown. C) The Western blot shows the degradation of RhoA as marker for C3 uptake and Rho-ADP-ribosylation. HT22 cells were treated with C3 (500 nM) alone or C3 (500 nM) plus vimentin (1 ng/µl) for 48 h. Cell lysates were submitted to Western blot analysis probing RhoA and β-actin. One representative Western blot experiment is shown (n = 3 independent experiments). D) Densitometric evaluation of RhoA (from C) and adjustment to the corresponding actin band are shown; the bars give the relative RhoA level. E) HT22 cells were incubated with C3 (500 nM) or C3 (500 nM) plus 1 ng/µl of either vimentin head-, rod- or tail-domain for 48 h. Cell lysates were submitted to Western blot analysis probing RhoA and β-actin. Decreased signal of RhoA reflects degradation of RhoA after ADP-ribosylation and thus, enhanced C3 uptake. One representative Western blot experiment is shown (n = 3 independent experiments). F) Densitometric evaluation of RhoA (from E) and adjustment to the corresponding actin band are shown; the bars give the relative RhoA level. G) J774A.1 macrophages were treated with C3 (500 nM) alone or C3 (500 nM) plus vimentin (1 ng/µl) for 2 h. Cell lysates were submitted to Western blot analysis probing RhoA and β-actin. One representative Western blot experiment is shown (n = 3 independent experiments). H) Primary astrocytes were exposed to C3 (300 nM) alone or a combination of C3 (300 nM) with different concentrations of vimentin (0.2, 2 and 20 ng/µl) for 6 h at 37°C. After incubation time the astrocytes were stained for the intermediate filament protein GFAP to visualize morphological changes.

Next the role of extracellular vimentin in C3 cell binding and uptake into intact HT22 cells was analyzed. HT22 cells were incubated with C3 or a combination of C3 with vimentin. Bound C3 was analyzed by Western blot analysis against anti-C3. Figure 4A and B show that binding of C3 depended on the concentration of vimentin. C3 binding decreased in the presence of high vimentin concentrations but slightly increased at low vimentin concentration.

To study the influence of vimentin on the uptake of C3, a concentration of 1 ng/µl of vimentin (whole protein) was used because this vimentin concentration seemed to have no effect on mere C3 binding. HT22 cells were incubated with C3 alone or with C3 plus vimentin. Cells were lysed and subjected to Western blot analysis against RhoA and β-actin (loading control). The combination of full length vimentin with C3 caused increased internalization as detected by ADP-ribosylation-induced RhoA degradation (Fig. 4C and D). The influence of vimentin fragments (head, rod and tail domain) on cell entry of C3 is shown in Figure 4E and F. Combination of C3 with vimentin rod domain resulted in the weakest Rho signal indicating advanced degradation of ADP-ribosylated Rho and, thus, enhanced uptake of C3. Vimentin alone had no effect on RhoA (Fig. 4E and F). To confirm this result, we additionally used the macrophage-like J774A.1 cells and established that added vimentin increased the C3 uptake in J774A.1 macrophages as determined by complete shift in the apparent molecular weight of ADP ribosylated RhoA in comparison to C3 alone (Fig. 4G). To check whether vimentin-mediated uptake is a general feature, C3 uptake into primary astrocytes were studied in the absence and presence of vimentin. Under the same experimental procedure as used for immortalized cultivated cells, C3 in combination with vimentin induced an enhanced stellated morphology of astrocytes compared to C3 in the absence of vimentin (Fig. 4H). In a previous study it was shown that C3 is taken up by astrocytes and ADP-ribosylated Rho GTPase, which results in a pronounced stellated morphology [22]. C3 in combination with vimentin (0.2 to 2 ng/µl) caused the strongest morphological changes whereas C3 alone induced only minor morphological changes and vimentin alone caused no changes. Thus, these results suggest that vimentin in general mediates C3 uptake into cells.

To rule out that added vimentin per se did not accelerate endocytosis, the influence of vimentin on the endocytosis of *Clostridium difficile* toxin A was studied; toxin A catalyzes the intracellular glucosylation of Rho, Rac and Cdc42 GTPases. HT22 cells were incubated with toxin A (100 ng/µl) alone or the combination of toxin A with full length vimentin. The kinetics of morphological changes and that of intracellular Rac1-glucosylation was identical in the presence and absence of vimentin (Fig. S4). Thus, vimentin does not per se accelerate toxin A endocytosis but specifically interacts with C3.

### Expression and distribution of vimentin in HT22 and J774A.1 cells

**Figure 5:**
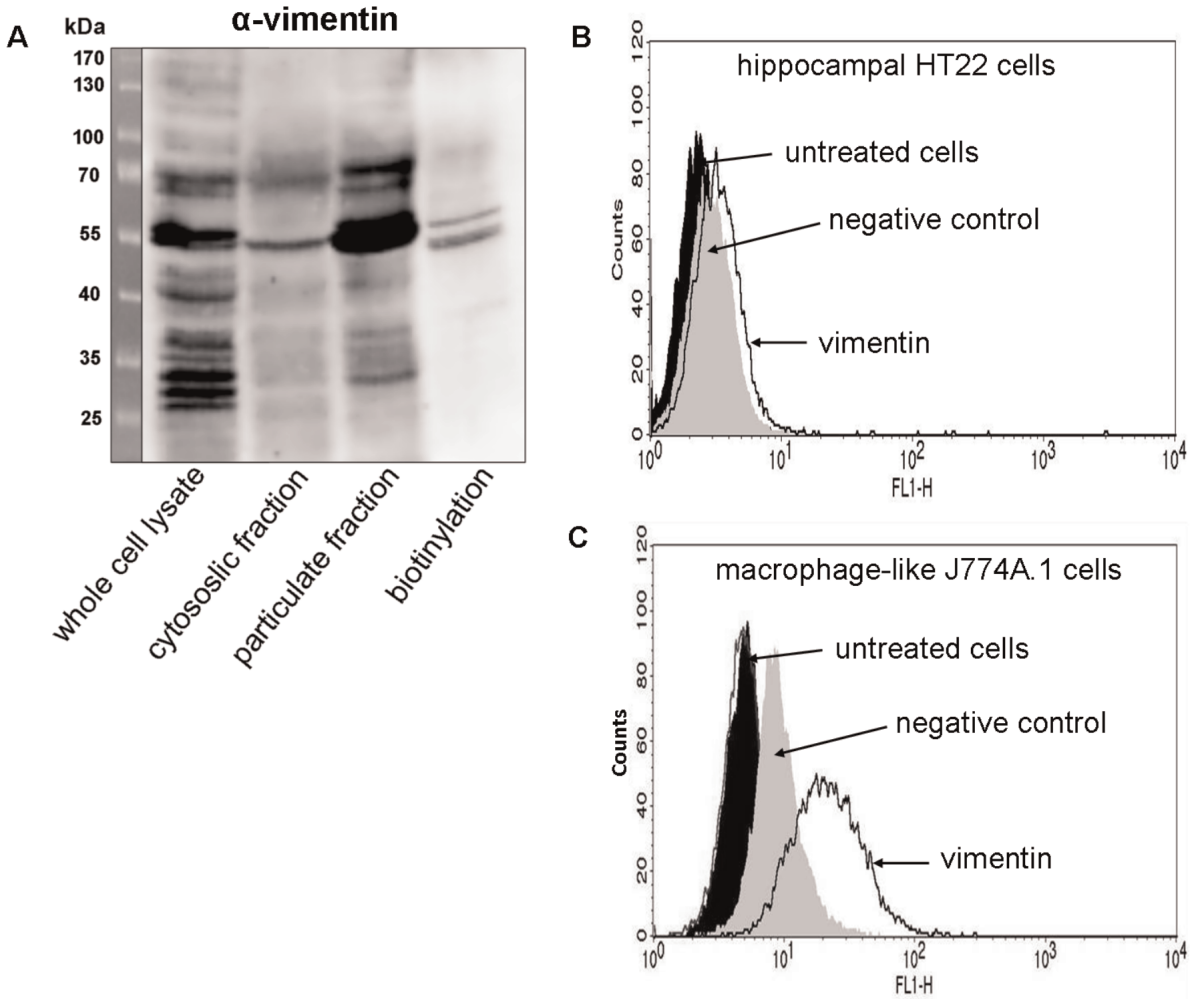
Vimentin is present at the cell surface of HT22 cells and J774A.1 macrophages. A) Intact HT22 cells were biotinylated for 1 h at 4°C. Whole cell lysates, cytosolic and particulate fractions were prepared. In addition, cell surface biotinylated proteins were enriched by precipitation with NeutrAvidin beads. The fractions and precipitation, respectively, were immunoblotted and probed with anti-vimentin. Biotinylation fraction represents the extracellular proteins exclusively. One representative Western blot experiment is shown (n = 3 independent experiments). Presence of vimentin at the cell surface of HT22 cells (B) and J774A.1 cells (C) was analyzed by FACS cytometry using anti-vimentin. Oregon green-488 conjugated goat anti-rabbit antibody alone served as negative control. Untreated cells were used as control.

Next the cell surface expression of vimentin was studied. As first step the monoclonal anti-vimentin V9 antibody was characterized using Western blot analysis of HT22 cell lysates and subfractions. As shown in Figure 5A, strong immune-reactive signals at 70 and 55 kDa and faint signals at 35–40 kDa were detected. Thus, anti-vimentin antibody recognized the genuine form of vimentin (55 kDa) in HT22 cells as well as different fragments and lower molecular weight degradation products, respectively. The 70 kDa band contains possibly modified vimentin such as the phosphorylated form. The broad range of band pattern with major bands at 55 kDa was already reported for the monoclonal anti-vimentin V9 antibody [34]. To check whether vimentin indeed was present at the cell surface of HT22 cells, a specific biotinylation of cell surface proteins were performed. Biotinylated surface proteins were captured by NeutrAvidin beads followed by separation on SDS-PAGE and vimentin Western blot analysis. Actually, vimentin was detected in the biotinylated sample (Fig. 5A). The biotinylated bands (55 kDa region) of the Western blot were subjected to mass spectrometry analysis, which clearly identified vimentin (Mascot score of 2324) (Fig. S5 and Table S3). To further verify the cell surface expression of vimentin, flow cytometry were performed. As illustrated (Fig. 5B) cell surface expression of vimentin at HT22 cells was detectable with the V9 clone anti-vimentin antibody directed against the tail domain. By flow cytometry, 4% of the cells were positive for surface vimentin staining. The amount of surface expressed vimentin was low but reproducible. To confirm this result, we repeated the flow cytometry analyses with the J774A.1 macrophages and observed a clear detection of 17% of cells stained for surface vimentin (Fig. 5C).

### Knock down of vimentin expression in HT22 and J774A.1 cells by siRNA

**Figure 6:**
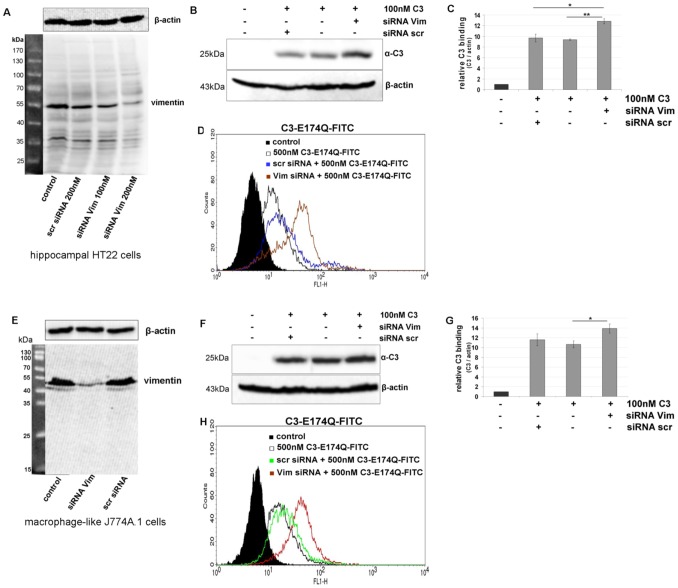
Knock down of vimentin in hippocampal HT22 cells and J774A.1 macrophages. A) HT22 cells were transfected with siRNA for 48 h (scr = scrambled, Vim = vimentin). Vimentin and β-actin were detected by Western blot analysis of cell lysates. B) After siRNA transfection for 48 h, HT22 cells were exposed to C3 (100 nM) for 1 h at 4°C. Bound C3 was detected in Western blot with anti-C3. β-actin was used as internal control. C) Densitometric evaluation of bound C3 (from B) and adjustment to the corresponding actin band are shown; the bars give the relative C3 binding. D) HT22 cells transfected with siRNA for 48 h were incubated with C3-E174Q-FITC (500 nM) for 1 h at 4°C and bound C3-E174Q-FITC was analyzed by FACS cytometry. E – G) Same experiments for J774A.1 macrophages. E) Knock down of vimentin. F) Binding of C3 to cells with vimentin knock down. G) Densitometric evaluation of F. H) Binding of C3-E174Q-FITC to cells with vimentin knock down and FACS analysis.

RNA interference technique was applied to further clear up the role of vimentin in C3 binding and uptake. Compared to control and scramble siRNA, vimentin siRNA transfection significantly reduced the expression of vimentin at protein level in HT22 cells (Fig. 6A) and J774A.1 cells (Fig. 6E). To check whether reduction in cellular vimentin concentration affected the interaction with C3, binding assays were performed 48 h after transfection. Surprisingly, efficacious knock down of vimentin resulted in an increased binding of C3, a finding which was reproduced by Western blot as well as by flow cytometry analysis (Fig. 6B, C and D for HT22 cells and Fig. 6F, G and H for J774A.1 cells).

**Figure 7:**
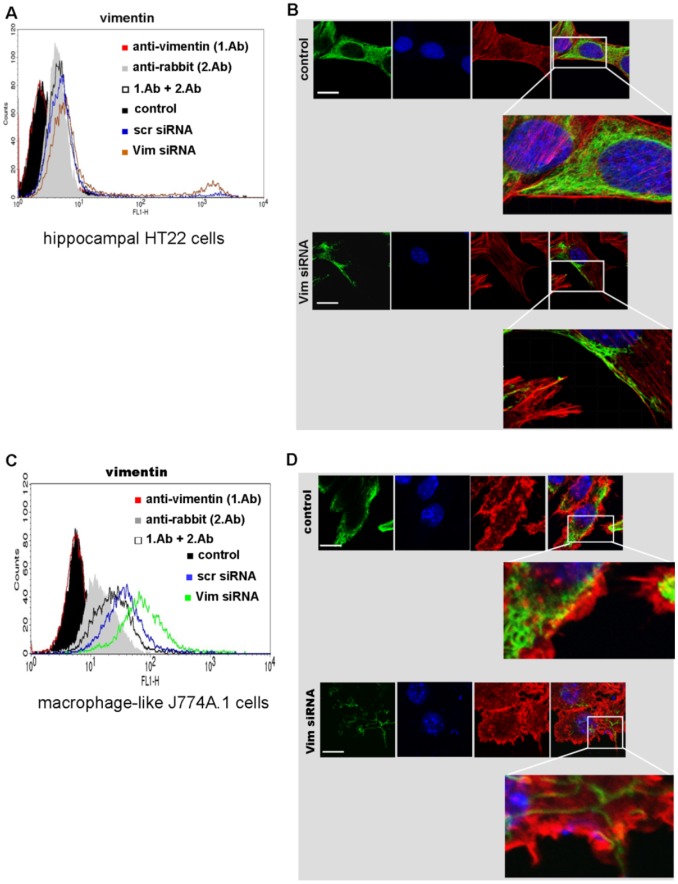
Analysis of vimentin distribution in analyzed cells. A) Vimentin was detected by anti-vimentin at the cell surface of HT22 cells transfected with siRNA for 48 h at 37°C by FACS analysis. B) Confocal microscopy of vimentin in HT22 cells transfected for 48 h at 37°C. The green (oregon green 488) anti-vimentin, DNA staining in blue (Dapi), rhodamine red-staining for actin and a merge image are shown for each panel. In the enlarged images the cell boundaries are shown. Significant difference between the vimentin distribution was detected for the transfected cells (lower panel) in comparison to the control (upper panel). Scale bar = 20 µM. C) Detection of vimentin at the cell surface of J774A.1 cells transfected with siRNA. D) Confocal microscopy of vimentin distribution in J774A.1 cells transfected for 48 h.

Cell surface expression of vimentin was checked 48 h after transfection; in fact, a slight increase of vimentin at the cell surface was detected (Fig. 7A). To study in detail whether and how siRNA transfection resulted in reduction and redistribution of vimentin, immunofluorescence microscopy analysis were performed. As illustrated in Figure 7B (bottom panel) Vim (vimentin) siRNA transfection for 48 h led to complete reduction and redistribution of vimentin. In control cells reticulate vimentin pervades the entire cytoplasmic region and the cell boundary was sharply defined (Fig. 7B, upper panel). In contrast, Vim siRNA transfected cells showed significantly reduced cytoplasmic vimentin. Different vimentin fragments were detectable and it seems that more vimentin fragments were at the cell periphery (Fig. 7B). As shown in Fig. 7C and D, same observations of vimentin distribution were done in Vim siRNA transfected J774A.1 cells. After siRNA knock down it seems that vimentin fragments protrude from the cell (Fig. 7D, bottom panel). A more detailed analysis of vimentin filament distribution after siRNA transfection is available as supplementary Figure S6. These findings support the hypothesis that destabilized tetrameric vimentin is incorporated into the membrane where degradation is delayed [35].

**Figure 8:**
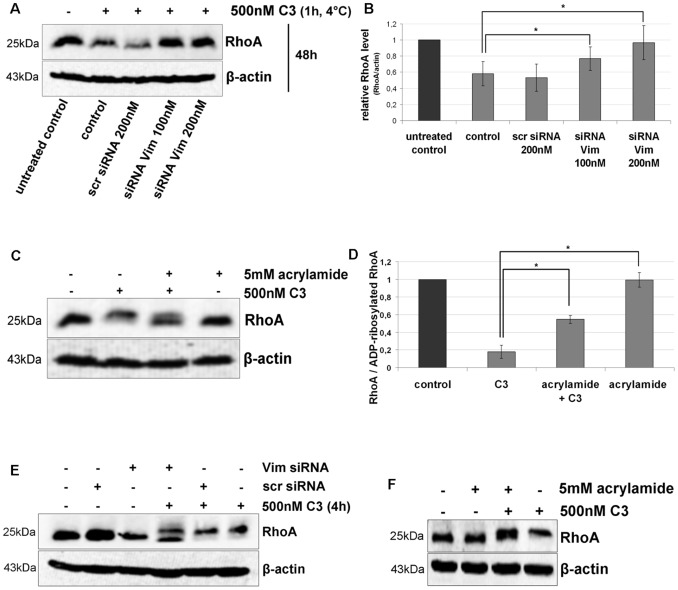
Uptake of C3 in HT22 and J744A.1 cells is dependent on vimentin distribution and integrity. A) Influence of Vim-siRNA knock down (for 48 h) on the uptake of C3 into HT22 cells detected as RhoA degradation (induced by C3-catalysed ADP-ribosylation). In a pulse-chase experiment, HT22 cells were incubated with C3 (500 nM) at 4°C for 60 min. Afterwards unbound C3 was removed by washing the cells three times with PBS and fresh medium was added. Cells were then cultivated for further 48 h. Cell lysates were generated and separated by SDS-PAGE followed by Western blot analysis probing RhoA and β-actin. One representative experiment is shown (n = 3 independent experiments). B) Cellular levels of RhoA proteins were quantified by densitometric evaluation of RhoA (from A) and adjusted to the corresponding actin band. C) HT22 cells were pre-treated with acrylamide (5 mM) for 30 min followed by incubation with C3 (500 nM) for 24 h. Cells were lysed and submitted to Western blot analysis probing RhoA and β-actin. C3 alone causes a complete mol weight shift of RhoA in SDS-PAGE. Western blot analysis of one representative experiment is shown (n = 3 independent experiments). D) RhoA shift (indicative of Rho-ADP-ribosylation) by quantified by densitometric evaluation of RhoA (from C) and adjusted to the corresponding β-actin signal. E) Influence of Vim-siRNA knock down (for 48 h) on the uptake of C3 into J774A.1 cells detected as incomplete RhoA ADP-ribosylation. J774A.1 macrophages were incubated with C3 (500 nM) at 37°C for 4 h. Cell lysates were generated and separated by SDS-PAGE followed by Western blot analysis probing RhoA and β-actin. One representative experiment is shown (n = 3 independent experiments). F) J774A.1 cells were pre-treated with acrylamide (5 mM) for 30 min followed by incubation with C3 (500 nM) for 4 h. Cells were lysed and submitted to Western blot analysis probing RhoA and β-actin.

Next the effect of vimentin knock down on the cellular uptake of C3 was studied. In a pulse-chase experiment exposure of HT22 cells to C3 caused degradation of RhoA as a marker for ADP-ribosylation. In Vim siRNA transfected cells C3- induced RhoA degradation is delayed (Fig. 8A and B), indicating a decreased uptake. The involvement of vimentin in both binding and uptake of C3 into HT22 cells was further confirmed by application of acrylamide. It has long been known that acrylamide at low concentrations causes collapse of intermediate filament structures but neither does affect microtubules nor actin filaments [36], [37]. In preceding experiments the effective but non-toxic concentration of acrylamide was determined; 5 mM of acrylamide caused vimentin disruption without affecting cell viability (cell rounding and decreased cell number as shown in Figure S7A and B). Furthermore, acrylamide did not affect C3 enzyme activity to ADP-ribosylate RhoA (Fig. S7C). Pretreatment of HT22 cells with acrylamide caused delayed ADP-ribosylation of RhoA (Fig. 8C and D). To confirm these results, we conducted further experiments with J774A.1 macrophages. First we investigated the effect of vimentin knock down on the C3 uptake. As shown in Figure 8E, vimentin knock down in J774A.1 cells caused a significant delayed ADP-ribosylation of RhoA as determined by double band of RhoA in comparison to complete mol shift by C3 alone (Fig. 8E). A similar result was obtained by acrylamide pre-treated cells. As seen by HT22 cells, pretreatment of J774A.1 macrophages with acrylamide delayed ADP-ribosylation of RhoA (Fig. 8F).

Taken together, these results strongly indicate that uptake of C3 in HT22 and J774A.1 cells is dependent on vimentin network.

## Discussion

C3 exoenzyme is still used as cell biological tool because of its high selective inactivation of RhoA/B/C GTPases in intact cells [8], [38], even in the era of knock-out techniques. C3 has an advantage over the application of RhoA-siRNA. RhoA-siRNA is accompanied by massive RhoB expression and activation [39]. This is based on the fact that the *rhoB* promoter is suppressed by RhoA and RhoA inactivation is always associated with RhoB expression [40]. C3, however, also causes strong RhoB expression but RhoB is completely inactivated by C3-catalyzed ADP-ribosylation. Thus, application of C3 represents the only approach to effectively inhibit RhoA without concomitant RhoB activation [41].

Poor cell accessibility has been overcome by generation of fusion constructs enhancing cell entry. However, some cell types are per se sensitive such as macrophages, neutrophils, astrocytes and neurons. C3 at nanomolar concentrations is able to efficiently enter the cells and cause ADP-ribosylation of Rho resulting in morphological changes [14], [23], [42]. The lack of cell entry domains of C3, as can be deduced from the crystal structure, makes it difficult to explain the process of internalisation. The first step of cell entry, however, is binding to membranous structures to trap the toxin at the cell surface and initiate cell entry. Thus, we started with the characterization and identification of the cellular C3 receptor.

Pre-tests revealed the proteineous nature of the putative receptor. Therefore, Western blot binding overlays were employed to identify distinct bands. Mass spectrometry analysis of one exemplarily SDS-PAGE band resulted in the identification of more than 100 proteins. To confine the receptor candidates the membrane extract was therefore resolved by 2D gel electrophoresis and the overlay was repeated after blotting. Now seven major spots (C3 binding to proteins) were visualized and identified by mass spectrometry. To further confine the candidates the inversed experiment was performed using C3 as immobilized bait. C3 bound proteins were again identified by mass spec. Now the lists of identified C3-binding proteins were compared and the intersection of both lists was determined. Two proteins, namely vimentin and β-actin, appeared in all identification approaches. Vimentin is an intermediate filament protein appearing in different posttranslational modifications and forms. Vimentin, the most abundant component of intermediate filaments is mainly involved in structural processes, such as wound healing [43] and is required for migration of peripheral blood mononuclear cells through the endothelial cell layer [44]. It is present in numerous different isoforms in the cell covering a molecular weight range between 42 and 60 kDa. Interestingly, it has been reported that vimentin is able to directly bind to *Pasteurella multicoda* toxin [45] and to *Salmonella* virulence protein SptP (Salmonella protein tyrosine phosphatase) [46]. Several reports show that vimentin to some extend is extracellularly located [26]–[30]. Moreover, vimentin possesses a GlcNAc-binding lectin-like property [47] and interacts when located at the cell surface with several pathogens such as porcine reproductive and respiratory syndrome virus (PRRSV) [48], *Escherichia coli* [49] or Japanese encephalitis virus [50]. Vimentin, a member of the type III intermediate filaments (IF), are detected in mesenchymal and some ectodermal cells during the early developmental stages. Recent studies have revealed vimentin as highly dynamic polymer, with three types of structures (granules, squiggles, and filaments) coexisting in the cell. Particularly vimentin IF varies in the form of granules and squiggles and have an important role in the assembly and disassembly of IF networks [51]. Vimentin is involved in many important physiological functions, such as signal transduction, gene regulation and distributions of organelles [52]. It shares a tripartite domain structure consisting of an N-terminal head domain, middle coiled-coil containing rod-domain, and a C-terminal tail domain [53]. Based on reliable identification and due to the reported presence at the cell surface (in contrast to actin), vimentin was selected for the further identification strategy.

The next step was to check whether vimentin is indeed present at the extracellular surface of HT22 cells. Two different approaches were applied: selective labeling of extracellular membrane proteins by biotinylation of intact cells, followed by an enrichment step and detection of vimentin by anti-vimentin in the fraction of biotinylated proteins. In addition, vimentin was identified in the immune-reactive band by mass spec. Second approach used the labeling of intact cells with anti-vimentin and analysis by flow cytometry. A small percentage of cells were in fact labeled with monoclonal anti-vimentin. Moreover, this result was confirmed by flow cytometry analysis of J774A.1 macrophages. HT22 cells revealed a much lower percentage of positive staining for surface vimentin expression in comparison to J774A.1 cell line. Thus, vimentin is present at the plasma membrane of intact HT22 and J774A.1 cells. Mor-Vaknin et al. reported on vimentin expression at the cell surface of activated macrophages and on vimentin secretion into the extracellular milieu [54]. Similar observations were found in astrocytes. Denosomin (1-deoxy-nor-sominone) stimulates astrocytes to secret vimentin as an axonal facilitator to increase axonal outgrowth in neurons [55]. Recently, surface-expressed forms of vimentin have been discovered in several cell lines [29], [30], [56]. For example, it was demonstrated that various prostate cancer cell lines like DU145, LNCaP and PC3 expressed two different domains of vimentin at the surface. On these cells, both the rod 1 coil domain and the C-terminal domain of vimentin were detected [57]. Notably, up-regulation of surface-located vimentin at injured skeletal muscle cells was recently shown to be a ligand for attachment of group A streptococci (GAS) [58]. Consistent with this view, we here show that vimentin was expressed at the cell surface of HT22 cells.

To investigate the role of vimentin in detail we used siRNA technology to knock down vimentin because we were able to detect vimentin by Western blot analysis in two described vimentin negative cancer cell lines like HT29 [59], [60] and A549 cells [61]. Indeed, many years ago it was demonstrated that various eukaryotic cell types start expressing vimentin upon culturing [62], [63]. Moreover, in vimentin knockout cells a changed cell shape [64], reduced mechanical stability with thicker actin bundles and less focal adhesions [43] and disruption of glial fibrillary acidic protein network [65] is observed. There are too many unknown parameters to characterize the vimentin dependent uptake of C3.

However, after siRNA vimentin knock down an enhanced binding of C3 in Western blot analysis and flow cytometry was observed. Confocal immunofluorescence microscopy of transfected cells detected different vimentin fragments at the cell periphery and flow cytometry revealed slight increase of vimentin at cell surface. Another study reports that vimentin undergoes phosphorylation resulting in disassembling of vimentin and expression of tetrameric vimentin at the cell surface [35]. Moreover, it was discussed that vimentin, rich in both hydrophilic and hydrophobic amino acid residues, may have affinity to lipid bilayers [66]. Therefore, tetrameric vimentin may move from the cytoplasm to the outer leaflet of the membrane. From our latter observations, we conclude that vimentin disassembling was induced by siRNA transfection and the fragments are either increasingly incorporated into the plasma membrane or degraded in a delayed manner. This hypothesis is supported by the observation that six days after transfection no increased C3 binding to Vim siRNA-transfected HT22 and J774A.1 cells is detectable anymore (Fig. S8). In this context, further studies have revealed, that vimentin is a highly dynamic polymer, with three types of intermediate structures - granules, squiggles, and longer filaments. The smaller subunits were found to be motile and showing movement preferentially towards peripheral regions of the cells [67], [68]. Other authors reported on an increased association of vimentin with the endoplasmic reticulum and the Golgi compartments, supporting the hypothesis that vimentin is secreted into the extracellular milieu via conventional secretory pathway [54]. The mechanism by which vimentin reaches the cell surface and the nature of domains exposed extracellularly remains to be investigated. The next step in studying the interaction of C3 with vimentin was to check whether binding to vimentin is functional that means whether it is involved in the uptake process. To this end recombinant vimentin and siRNA technique was applied. Surprisingly, pre-incubation of C3 with vimentin resulted in a bi-phasic effect: at low concentration binding was increased whereas at high concentration binding was reduced. One explanation is that at high concentration of vimentin probably supports oligomer formation resulting in binding of C3 to these vimentin oligomers instead to the cell surface. In low concentrations vimentin is monomeric thereby facilitating binding of C3 to the membranes. Interestingly, there seems to be a neutral concentration of vimentin (1 ng/µl), which does not affect binding of C3 to HT22 cells and J774A.1 macrophages. In contrast, siRNA knock down of vimentin resulted in a delayed uptake of C3 detected by the absence of degradation of ADP-ribosylated RhoA in HT22 cells and in incomplete shift of ADP-ribosylated RhoA in J774A.1 macrophages. Six days after siRNA transfection this effect was abolished and the amount of vimentin was comparable with control cells (Fig. S8). Furthermore, disruption of vimentin network through acrylamide causes delayed RhoA ADP-ribosylation suggesting that C3 uptake in analyzed cells is dependent on vimentin. This finding is supported by the observation that the combination of vimentin and C3 induced in primary astrocytes a pronounced morphological change as compared to C3 or vimentin alone. In agreement with our findings, several studies show that vimentin is involved in endocytotic uptake of pathogens such as human immunodeficiency virus type 1 [69], vaccinia virus [70], and human T-cell leukemia virus type I [71]. For example, picornaviruses recruit vimentin as a component of the cellular attachment mechanism, suggesting a role for surface vimentin for cellular entry [72]. Vimentin is also necessary for trafficking of blue tongue virus to the cell surface [73].

Vimentin is a three domain protein consisting of the head (aa1–101), rod (aa102–410) and tail (aa411–466) domain. Applying overlay technique we showed that C3 exclusively binds to the rod domain. Vimentin reportedly interacts with various proteins (see Table S4). However, the binding domains of vimentin have only been determined in a few of interactions and further analysis will be required to explore the exact molecular mechanism of protein-vimentin interaction.

It is known that vimentin is initially expressed in neuronal precursors where expression is necessary for neurite extension. It is replaced by neurofilaments shortly after the immature neurons become post-mitotic. In adult brain, vimentin expression is largely restricted to vascular endothelial cells and certain subpopulations of glial cells [74]. An abundant vimentin expression is observed in adult neurons in response to injury [75]. For example, immediately after slicing, neurons generally lacked detectable vimentin expression. When cells in brain slices were allowed to recover in culture for 3–16 h, many neurons in the cerebral cortex and hippocampus exhibit abundant vimentin expression [75]. In this context, in a rat sciatic nerve crush model it was shown that vimentin expression is elevated in sciatic nerve axoplasm after injury [76]. Thus, it is conceivable that vimentin is expressed in neuronal lesion models and mediates C3 binding and uptake. This may explain why C3 and its enzyme-deficient form are able to act on neurons triggering recovery in peripheral and central neuronal lesion models [77], [78]. Moreover, this hypothesis is supported by the observation of axonotrophic function of C3 and enzyme-deficient C3-E174Q in cultivated peripheral sensory neurons dissociated from adult dorsal root ganglion of the rat [79]. In contrast, in organotypic spinal cord preparations a reduction of axonal growth of motoneurons and no effect on sensory axon outgrowth from whole dorsal root ganglia (DRG) explants by C3 were demonstrated [80]. It is possible that cultivation of sensory neurons caused abundant vimentin expression and therefore enhanced C3 induced uptake. However, the first study revealed that an activation of extracellular signal-regulated kinase (ERK) and Akt signaling pathways were involved in axon regeneration of adult sensory neurons. Notably, after nerve lesion vimentin is upregulated and binds to phosphorylated Erk and mediates retrograde transport of pErk in the cell body of dorsal root ganglia [81]. These findings suggest that vimentin is involved in C3 binding and uptake and is important for inducing of axonotrophic effects.

In conclusion, our findings strongly indicate that vimentin is expressed at the cell surface of hippocampal HT22 cells and J744A.1 macrophages and gets internalized upon binding to C3. After binding to surface rod-domain of vimentin C3 is endocytosed via vimentin-mediated endocytosis. Further analysis will be required to explore the exact molecular mechanism of C3-vimentin interaction. Because C3 lacks a translocation domain, demonstration of the interaction of C3 either with vimentin at cell surface or uptake and subsequent association of C3 with the cellular intermediate filament protein vimentin represent important steps in understanding how C3 may access its intracellular target Rho. The nature of this interaction is not fully elucidated but represents a starting point for future studies on the property of vimentin as a target structure for bacterial toxins.

## Supporting Information

Figure S1 **Pronase treatment resulted in cell detachment.** HT22 cells were treated with 500 µg/ml of pronase for different incubation times at 4°C. The detached cells were analysed microscopically. Serum was added to detached cells to block pronase activity and cells were then washed with PBS and reseeded for 24 h at 37°C in media with 5% FCS.
(TIF)

Figure S2 **Binding of C3 and C3-E174Q to HT22 cells.** HT22 cells were exposed to increasing concentrations of C3 ore C3-E174Q for 1 h at 4°C. Subsequently, C3bot and β-actin were detected by Western blot analysis. Densitometric quantification bound C3. All signal intensities of C3 were adjusted to the intensity of the corresponding β-actin signal. The differences in results were not statistically significant indicating that binding of C3 and C3-E174Q to HT22 cells is completing comparable.
(TIF)

Figure S3 **2D-overlay experiments.** Particulate fraction of HT22 cells were separated by 2D gel electrophoresis in the pH range 4–8, transferred to PVDF membrane and overlaid with C3 followed by immunoblotting and probing bound C3. Indicated positive spots in the 40–70 kDa region were in-gel digested with trypsin and subjected to mass spectrometry (spot 1 and 2 = HSP7C, spot 3 = vimentin, spot 4 = HNRPF, spot 5 = actin, spot 6 and 7 = nucleophosmin; Table S1, showing results for each spot).
(TIF)

Figure S4 **Effects in HT22 cells treated with Toxin A or Toxin A together with vimentin for the indicated times.** A) Toxin A (100 ng/ml) induced morphological changes in HT22 cells within 3 h and this effect was more pronounced with longer incubation time. The combination of vimentin and Toxin A did not result in increase of morphological changes or faster cell rounding in comparison to Toxin A alone. Scale bar = 100 µM. B) The Rac glucosylation, which was assayed (after different time points as indicated) using a monoclonal anti-Rac1 antibody (Clone 102, BD Transduction Laboratories, Heidelberg, Germany), was in strong correlation with cell rounding (Figure S3A).
(TIF)

Figure S5 **Schematic representation of protein identification with gel-based separation.** Whole HT22 cell lysate, particulate fraction and biotin-labeled cell surface proteins were separated by 10% SDS-PAGE and stained with coomassie brilliant blue. The 55 kDa band of biotinylated sample was digested with trypsin and peptides were subjected to mass spectrometry analysis (Table S3, showing results of analysis).
(TIF)

Figure S6 **Detailed analysis of vimentin distribution after siRNA transfection.** A) Results of hippocampal HT22 cells. In the middle of the panel were shown the immunhistochemical analysis as indicated. The quantification of vimentin intensity was done with ImageJ software. ImageJ creates a surface plot from all pixel intensities of vimentin to visualize the distribution of vimentin. Additionally, ImageJ calculates a grey level histogram of the image. In the histogram the x-axis represents the grey values and the y-axis shows the number of pixels. The histogram displays the distribution of grey values which correspondents to vimentin intensity (the lighter the gray value the higher the vimentin intensity).
(TIF)

Figure S7 **Effects of acrylamide on vimentin network and C3 enzyme activity.** A) Morphological changes in HT22 cells exposed with 5 mM acrylamide for indicated times. Scale bar = 100 µM. B) Immunostaining with anti-vimentin mAb V9 and immunfluorescence microscopy was performed to study the distribution of vimentin filaments in acrylamide treated cells. The green anti-vimentin staining shows disruption in vimentin network in acrylamide treated cells. Scale bar = 20 µM. C) To rule out that acrylamide does not affect C3 enzyme activity ADP-ribosylation was performed in contemporary of acrylamide. Acrylamide pre-incubated HT22 cells were lysed. Subsequently, cell lysates were exposed to 1 µM C3 and 1 µCi [^32^P]NAD (Amersham Life Sciences, Arlington Heights, IL, USA) in 20 µl of 4× buffer containing 50 mM HEPES (pH 7.3), 10 mM MgCl2, 10 mM dithiothreitol, 10 mM thymidine and 10 µM NAD at 37°C for 5 or 10 min. The reaction was terminated by addition of Laemmli sample buffer, and then incubated at 95°C for 10 min. Samples were resolved by 15% SDS-PAGE, and the ADP-ribosylated Rho was analyzed by phosphorimaging (Cyclone, Packard American Instrument, MA, USA).
(TIF)

Figure S8 **Binding of C3 to HT22 and J774A.1 cells 6 days after siRNA transfection.** HT22 cells (A) and J774A.1 cells (C) were transfected with siRNA (scr = scrambled, Vim = vimentin). 144 h later Vimentin and β-actin were detected by Western blot analysis of cell lysates. 144 h after siRNA transfection, HT22 cells (B) and J774A.1 cells (D) were exposed to C3 (100 nM) for 1 h at 4°C. Bound C3 was detected in Western blot with anti-C3. β-actin was used as internal control.
(TIF)

Table S1 **Identification of C3 interacting proteins by two-dimensional electrophoresis followed by C3-overlay and LC-MS/MS analysis.** Results are LC-MS/MS data processed with Mascot search engine and the Swissprot database.
(DOC)

Table S2 **Complete list of proteins bound to C3, including accession No, Mascot detection score, number of identified peptides, mass, isoelectric point and sequence coverage.** Results are LC-MS/MS data processed with Mascot search engine and the Swissprot database.
(DOC)

Table S3 **Identified proteins in the biotinylated samples.** Results are LC-MS/MS data processed with Mascot search engine and the Swissprot database.
(DOC)

Table S4 **Proteins which interact with vimentin.** n.d. = not described.
(DOC)

## References

[1] Aktories K, Frevert J (1987) ADP-ribosylation of a 21–24 kDa eukaryotic protein(s) by C3, a novel botulinum ADP-ribosyltransferase, is regulated by guanine nucleotide. Biochem J 247: 363–368. 3122724 10.1042/bj2470363 PMC1148417

[2] Just I, Selzer J, Jung M, van Damme J, Vandekerckhove J, et al (1995) Rho-ADP-ribosylating exoenzyme from *Bacillus cereus*. Purification, characterization, and identification of the NAD-binding site. Biochemistry 34: 334–340 10.1021/bi00001a041 7819216

[3] Wilde C, Chhatwal GS, Schmalzing G, Aktories K, Just I (2001) A novel C3-like ADP-ribosyltransferase from *Staphylococcus aureus* modifying RhoE and Rnd3. J Biol Chem 276: 9537–42 10.1074/jbc.M011035200 11124969

[4] Sehr P, Joseph G, Genth H, Just I, Pick E, et al (1998) Glucosylation and ADP-ribosylation of Rho proteins: Effects on nucleotide binding, GTPase activity, and effector coupling. Biochemistry 37: 5296–5304 10.1021/bi972592c 9548761

[5] Genth H, Gerhard R, Maeda A, Amano M, Kaibuchi K, et al (2003) Entrapment of rho ADP-ribosylated by *Clostridium botulinum* C3 exoenzyme in the Rho-guanine nucleotide dissociation inhibitor-1 complex. J Biol Chem 278: 28523–28527 10.1074/jbc.M301915200 12750364

[6] Wiegers W, Just I, Müller H, Hellwig A, Traub P, et al (1991) Alteration of the cytoskeleton of mammalian cells cultured in vitro by *Clostridium botulinum* C2 toxin and C3 ADP-ribosyltransferase. Eur J Cell Biol 54: 237–245. 1908779

[7] Paterson HF, Self AJ, Garrett MD, Just I, Aktories K, et al (1990) Microinjection of recombinant p21rho induces rapid changes in cell morphology. J Cell Biol 111: 1001–1007 10.1083/jcb.111.3.1001 2118140 PMC2116288

[8] Aktories K, Just I (2005) Clostridial Rho-inhibiting protein toxins. Curr Top Microbiol Immunol 291: 113–145 10.1007/3-540-27511-87 15981462

[9] Genth H, Dreger SC, Huelsenbeck J, Just I (2008) *Clostridium difficile* toxins: more than mere inhibitors of Rho proteins. Int J Biochem Cell Biol 40: 592–597 10.1016/j.biocel.2007.12.014 18289919

[10] Ahnert-Hilger G, Höltje M, Grosse G, Pickert G, Mucke C, et al (2004) Differential effects of Rho GTPases on axonal and dendritic development in hippocampal neurones. J Neurochem 90: 9–18 10.1111/j.1471-4159.2004.02475.x 15198662

[11] Sandvig K, Skotland T, van Deurs B, Klokk TI (2013) Retrograde transport of protein toxins through the Golgi apparatus. Histochem Cell Biol 140: 317–26 10.1007/s00418-013-1111-z 23765164

[12] Han S, Arvai AS, Clancy SB, Tainer JA (2001) Crystal structure and novel recognition motif of rho ADP-ribosylating C3 exoenzyme from *Clostridium botulinum*: structural insights for recognition specificity and catalysis. J Mol Biol 305: 95–107 10.1006/jmbi.2000.4292 11114250

[13] Fahrer J, Kuban J, Heine K, Rupps G, Kaiser E, et al (2010) Selective and specific internalization of clostridial C3 ADP-ribosyltransferases into macrophages and monocytes. Cell Microbiol 12: 233–47 10.1111/j.1462-5822.2009.01393.x 19840027

[14] Rotsch J, Rohrbeck A, May M, Kolbe T, Hagemann S, et al (2012) Inhibition of macrophage functions by *C. botulinum* exoenzyme C3. Naunyn Schmiedebergs Arch Pharmacol 385: 883–90 10.1007/s00210-012-0764-9 22644106

[15] Hoffmann A, Hofmann F, Just I, Lehnardt S, Hanisch UK, et al (2008) Inhibition of Rho-dependent pathways by Clostridium botulinum C3 protein induces a proinflammatory profile in microglia. Glia 56: 1162–75 10.1002/glia.20687 18442097

[16] Geny B, Popoff MR (2006) Bacterial protein toxins and lipids: role in toxin targeting and activity. Biol Cell 98: 633–51 10.1042/BC20060038 17042741

[17] Holmgren J, Mansson JE, Svennerholm L (1974) Tissue receptor for cholera exotoxin: structural requirements of G_M1_, ganglioside in toxin binding and inactivation. Med Biol 52: 229–233. 4214479

[18] Kitamura M, Iwamori M, Nagai Y (1980) Interaction between *Clostridium botiulinum* neurotoxin and gangliosides. Biochim Biophys Acta 628 328–335: doi.org/10.1016/0304–4165(80)90382-7. 10.1016/0304-4165(80)90382-7 6768400

[19] Fishman PH (1982) Role of membrane gangliosides in the binding and action of bacterial toxins. J Membr Biol 69: 85–97 10.1007/BF01872268 6752418

[20] Merritt EA, Zhang Z, Pickens JC, Ahn M, Hol WG, et al (2002) Characterization and crystal structure of a high-affinity pentavalent receptor-binding inhibitor for cholera toxin and *E. coli* heat-labile enterotoxin. J Am Chem Soc 124: 8818–24 10.1021/ja0202560 12137534

[21] Tobaben S, Grohm J, Seiler A, Conrad M, Plesnila N, et al (2011) Bid-mediated mitochondrial damage is a key mechanism in glutamate-induced oxidative stress and AIF-dependent cell death in immortalized HT-22 hippocampal neurons. Cell Death Differ. 2011 Feb;18 (2): 282–92 10.1038/cdd.2010.92 PMC3131888 20689558

[22] Höltje M, Hoffmann A, Hofmann F, Mucke C, Grosse G, et al (2005) Role of Rho-GTPase in astrocyte morphology and migratory response during *in vitro* wound healing. J Neurochem 5: 1237–48 10.1111/j.1471-4159.2005.03443.x 16150054

[23] Rohrbeck A, Kolbe T, Hagemann S, Genth H, Just I (2012) Distinct biological activities of C3 and ADP-ribosyltransferase-deficient C3-E174Q. FEBS 279: 2657–71 10.1111/j.1742-4658.2012.08645 22621765

[24] Luecke N, Templin C, Muetzelberg MV, Neumann D, Just I, et al (2010) Secreted proteome of the murine multipotent hematopoietic progenitor cell line DKmix. Rapid Commun Mass Spectrom 24: 561–70 10.1002/rcm.4412 20127908

[25] Liang JJ, Yu CY, Liao CL, Lin YL (2011) Vimentin binding is critical for infection by the virulent strain of Japanese encephalitis virus. Cell Microbiol 13: 1358–70 10.1111/j.1462-5822.2011.01624.x 21707907

[26] Podor TJ, Singh D, Chindemi P, Foulon DM, McKelvie R, et al (2002) Vimentin exposed on activated platelets and platelet microparticles localizes vitronectin and plasminogen activator inhibitor complexes on their surface. J Biol Chem. 277: 7529–39 10.1074/jbc.M109675200 11744725

[27] Boilard E, Bourgoin SG, Bernatchez C, Surette ME (2003) Identification of an autoantigen on the surface of apoptotic human T cells as a new protein interacting with inflammatory group IIA phospholipase A2.Blood. 102: 2901–9 10.1182/blood-2002-12-3702 12829607

[28] Xu B, deWaal RM, Mor-Vaknin N, Hibbard C, Markovitz DM, et al (2004) The endothelial cell-specific antibody PAL-E identifies a secreted form of vimentin in the bloodvasculature. Mol Cell Biol. 24: 9198–206 10.1128/MCB.24.20.9198-9206.2004 PMC517872 15456890

[29] Moisan E, Girard D (2006) Cell surface expression of intermediate filament proteins vimentin and lamin B1 in human neutrophil spontaneous apoptosis. J Leukoc Biol 79: 489–98 10.1189/jlb.0405190 16365157

[30] Huet D, Bagot M, Loyaux D, Capdevielle J, Conraux L, et al (2006) SC5 mAb represents a unique tool for the detection of extracellular vimentin as a specific marker of Sezary cells. J Immunol 176: 652. 16365461 10.4049/jimmunol.176.1.652

[31] Faigle W, Colucci-Guyon E, Louvard D, Amigorena S, Galli T (2000) Vimentin filaments in fibroblasts are a reservoir for SNAP23, a component of the membrane fusion machinery. Mol Biol Cell. 11: 3485–94 10.1091/mbc.11.10.3485 PMC15008 11029050

[32] Styers ML, Salazar G, Love R, Peden AA, Kowalczyk AP, et al (2004) The endo-lysosomal sorting machinery interacts with the intermediate filament cytoskeleton. Mol Biol Cell. 15: 5369–82 10.1182/blood-2002-12-3702 PMC532017 15456899

[33] Walter M, Chen FW, Tamari F, Wang R, Ioannou YA (2009) Endosomal lipid accumulation in NPC1 leads to inhibition of PKC, hypophosphorylation of vimentinand Rab9 entrapment. Biol Cell. 101: 141–52 10.1042/BC20070171 18681838

[34] Kim JK, Fahad AM, Shanmukhappa K, Kapil S (2006) Defining the cellular target(s) of porcine reproductive and respiratory syndrome virus blocking monoclonal antibody 7G10. J Virol 80: 689–696 10.1128/JVI.80.2.689-696.2006 16378972 PMC1346842

[35] Ise H, Goto M, Komura K, Akaike T (2012) Engulfment and clearance of apoptotic cells based on a GlcNAc-binding lectin-like property of surface vimentin. Glycobiology 22: 788–805 10.1093/glycob/cws052 22345628

[36] Eckert BS (1985) Alteration of intermediate filament distribution in PtK1 cells by acrylamide. Eur. J Cell Biol 37: 169–74. 2411559

[37] Haudenschild DR, Chen J, Pang N, Steklov N, Grogan SP, et al (2011) Vimentin contributes to changes in chondrocyte stiffness in osteoarthritis. J Orthop Res 29: 20–5 10.1002/jor.21198 20602472 PMC2976780

[38] Just I, Boquet P (2000) Large clostridial cytotoxins as tools in cell biology. Curr Top Microbiol Immunol 250: 97–107. 10981359 10.1007/978-3-662-06272-2_5

[39] Ho TT, Merajver SD, Lapière CM, Nusgens BV, Deroanne CF (2008) RhoA- GDP regulates RhoB protein stability. Potential involvement of RhoGDI-alpha. J Biol Chem 283: 21588–98 10.1074/jbc.M710033200 18524772

[40] Huelsenbeck J, Dreger SC, Gerhard R, Fritz G, Just I, et al (2007) Upregulation of the immediate early gene product RhoB by exoenzyme C3 from *Clostridium limosum* and toxin B from *Clostridium difficile* . Biochemistry 46: 4923–31 10.1021/bi602465z 17397186

[41] Just I, Rohrbeck A, Huelsenbeck SC, Hoeltje M (2011) Therapeutic effects of *Clostridium botulinum* C3 exoenzyme. Naunyn Schmiedebergs Arch Pharmacol 383: 247–52 10.1007/s00210-010-0589-3 21193903

[42] Höltje M, Just I, Ahnert-Hilger G (2011) Clostridial C3 proteins: recent approaches to improve neuronal growth and regeneration. Ann Anat 193: 314–20. Review. doi:10.1016/j.aanat.2011.01.008. 21459564

[43] Eckes B, Dogic D, Colucci-Guyon E, Wang N, Maniotis A, et al (1998) Impaired mechanical stability, migration and contractile capacity in vimentin-deficient fibroblasts. J Cell Sci 111: 1897. 9625752 10.1242/jcs.111.13.1897

[44] Nieminen M, Henttinen T, Merinen M, Marttila-Ichihara F, Eriksson JE, et al (2006) Vimentin function in lymphocyte adhesion and transcellular migration. Nat Cell Biol 8: 156 10.1038/ncb1355 16429129

[45] Shime H, Ohnishi T, Nagao K, Oka K, Takao T, et al (2002) Association of *Pasteurella multocida* toxin with vimentin. Infect Immun 70: 6460–3 10.1128/IAI.70.11.6460-6463.2002 12379728 PMC130396

[46] Murli S, Watson RO, Galan JE (2001) Role of tyrosine kinases and the tyrosine phosphatase SptP in the interaction of Salmonella with host cells. Cell Microbiol 3: 795–810 10.1046/j.1462-5822.2001.00158.x 11736992

[47] Ise H, Kobayashi S, Goto M, Sato T, Kawakubo M, et al (2010) Vimentin and desmin possess GlcNAc-binding lectin-like properties on cell surfaces. Glycobiology 20: 843–864 10.1093/glycob/cwq039 20332081

[48] Kim JK, Fahad AM, Shanmukhappa K, Kapil S (2006) Defining the cellular target(s) of porcine reproductive and respiratory syndrome virus blocking monoclonal antibody 7G10. J Virol 80: 689–696 10.1128/JVI.80.2.689-696.2006 16378972 PMC1346842

[49] Zou Y, He L, Huang SH (2006) Identification of a surface protein on human brain microvascular endothelial cells as vimentin interacting with *Escherichia coli* invasion protein IbeA. Biochem Biophys Res Commun 351: 625–630 10.1016/j.bbrc.2006.10.091 17083913

[50] Das S, Ravi V, Desai A (2011) Japanese encephalitis virus interacts with vimentin to facilitate its entry into porcine kidney cell line. Virus Research 160: 404–408 10.1016/j.virusres.2011.06.001 21798293

[51] Chou YH, Khuon S, Herrmann H, Goldman RD (2003) Nestin promotes the phosphorylation-dependent disassembly of vimentin intermediate filaments during mitosis. Mol Biol Cell 14: 1468–1478 10.1091/mbc.E02-08-0545 12686602 PMC153115

[52] Ivaska J, Pallari HM, Nevo J, Eriksson JE (2007) Novel functions of vimentin in cell adhesion, migration, and signaling. Exp Cell Res 313 2050–2062: doi.org/10.1016/j.yexcr.2007.03.040. 17512929

[53] Chernyatina AA, Nicolet S, Aebi U, Herrmann H, Strelkov SV (2013) Atomic structure of the vimentin central α-helical domain and its implications for intermediate filament assembly. PNAS 109: 13620–5 10.1073/pnas.1206836109 PMC3427084 22869704

[54] Mor-Vaknin N, Punturieri A, Sitwala K, Markovitz DM (2003) Vimentin is secreted by activated macrophages. Nat Cell Biol 5: 59 10.1038/ncb898 12483219

[55] Teshigawara K, Kuboyama T, Shigyo M, Nagata A, Sugimoto K, et al (2013) A novel compound, denosomin, ameliorates spinal cord injury via axonal growth associated with astrocyte-secreted vimentin. Br J Pharmacol 168: 903–919 10.1111/j.1476-5381.2012.02211.x 22978525 PMC3631379

[56] Bhattacharya R, Gonzalez AM, Debiase PJ, Trejo HE, Goldman RD, et al (2009) Recruitment of vimentin to the cell surface by beta3 integrin and plectin mediates adhesion strength. J Cell Sci 122: 1390–400 10.1242/jcs.043042 19366731 PMC2721003

[57] Steinmetz NF, Maurer J, Sheng H, Bensussan A, Maricic I, et al (2011) Two domains of Vimentin Are expressed on the Surface of Lymph Node, Bone and Brain Metastatic Prostate Cancer Lines along with the Putative Stem Cell Marker Proteins CD44 and CD133. Cancers 3: 2870–2885 10.3390/cancers3032870 24212937 PMC3759176

[58] Bryant AE, Bayer CR, Huntington JD, Stevens DL (2006) Group A streptococcal myonecrosis: increased vimentin expression after skeletal-muscle injury mediates the binding of *Streptococcus pyogenes* . J Infect Dis 193: 1685–1692 10.1086/504261 16703512

[59] Mitchell BS, Horny HP, Schumacher U (1997) Immunophenotyping of human HT29 colon cancer cell primary tumours and their metastases in severe combined immunodeficient mice. Histochem J 29: 393–9. 9184853 10.1023/a:1026490901926

[60] Lahat G, Zhu QS, Huang KL, Wang S, Bolshakov S, et al (2010) Vimentin is a novel anti-cancer therapeutic target; insights from in vitro and in vivo mice xenograft studies. PLoS One. 5(4): e10105 10.1371/journal.pone.0010105 PMC2855704 20419128

[61] Park SM, Gaur AB, Lengyel B, Peter ME (2008) The miR-200 family determines the epithelial phenotype of cancer cells by targeting the E-cadherin repressors ZEB1 and ZEB2. Genes Dev 22: 894–907 10.1101/gad.1640608 18381893 PMC2279201

[62] Rittling SR, Coutinho L, Amram T, Kolbe M (1989) AP-1/jun binding sites mediate serum inducibility of the human vimentin promoter. Nucleic Acids Res 17: 1619–33. 2922288 10.1093/nar/17.4.1619 PMC336896

[63] Pieper FR, Van de Klundert FA, Raats JM, Henderik JB, Schaart G, et al (1992) Regulation of vimentin expression in cultured epithelial cells. Eur J Biochem 210: 509–19. 1459133 10.1111/j.1432-1033.1992.tb17449.x

[64] Mendez MG, Kojima S, Goldman RD (2010) Vimentin induces changes in cell shape, motility, and adhesion during the epithelial to mesenchymal transition. FASEB 24: 61838–1851. 10.1096/fj.09-151639 PMC2874471 20097873

[65] Galou M, Gao J, Humbert J, Mericskay M, Li Z, Paulin D, et al (1997) The importance of intermediate filaments in the adaptation of tissues to mechanical stress: Evidence from gene knockout studies. Biology of Cell 89: 85–97. 9351189

[66] Perides G, Harter C, Traub P (1987) Electrostatic and hydrophobic interactions of the intermediate filament protein vimentin and its amino terminus with lipid bilayers. J Biol Chem 262: 13742–9. 3308882

[67] Goldman RD, Khuon S, Chou YH, Opal P, Steinert PM (1996) The function of intermediate filaments in cell shape and cytoskeletal integrity. J Cell Biol 134: 971–83 10.1083/jcb.134.4.971 8769421 PMC2120965

[68] Chou YH, Goldman RD (2000) Intermediate filaments on the move. J Cell Biol 150: F101–6 10.1083/jcb.150.3.F101 10931880 PMC2175194

[69] Thomas EK, Connelly RJ, Pennathur S, Dubrovsky L, Haffar OK, et al (1996) Anti-idiotypic antibody to the V3 domain of gp120 binds to vimentin: a possible role of intermediate filaments in the early steps of HIV-1 infection cycle. Viral Immunol 9: 73–87 10.1089/vim.1996.9.73 8822624

[70] Risco C, Rodriguez JR, Lopez-Iglesias C, Carrascosa JL, Esteban M, et al (2002) Endoplasmic reticulum-Golgi intermediate compartment membranes and vimentin filaments participate in vaccinia virus assembly. J Virol 76: 1839–1855 10.1128/JVI.76.4.1839-1855.2002 11799179 PMC135913

[71] Lilienbaum A, Duc Dodon M, Alexandre C, Gazzolo L, Paulin D (1990) Effect of human T-cell leukemia virus type I tax protein on activation of the human vimentin gene. J. Virol. 64, 256–263. 10.1128/jvi.64.1.256-263.1990 PMC249098 2293664

[72] Kristopher J, Destito G, Plummer EM, Trauger SA, Siuzdak G, et al (2009) Endothelial Targeting of Cowpea Mosaic Virus (CPMV) via Surface Vimentin. PloS Pathogens 5: e1000417 10.1371/journal.ppat.1000417 19412526 PMC2670497

[73] Bhattacharya B, Noad RJ, Roy P (2007) Interaction between Bluetongue virus outer capsid protein VP2 and vimentin is necessary for virus egress. Virology of Journal 4: 7 10.1186/1743-422X-4-7 PMC1783847 17224050

[74] Izmiryan A, Franco CA, Paulin D, Li Z, Xue Z (2009) Synemin isoforms during mouse development: multiplicity of partners in vascular and neuronal systems. Exp Cell Res 315: 769–783 10.1007/s11064-009-0111-9 19124017

[75] Levin EC, Acharya NK, Sedeyn JC, Venkataraman V, D’Andrea MR, et al (2009) Neuronal expression of vimentin in the Alzheimer’s disease brain may be part of a generalized dendritic damage-response mechanism. Brain Res 1298: 194–207 10.1016/j.brainres.2009.08.072 19728994

[76] Perlson E, Michaelevski I, Kowalsman N, Ben-Yaakov K, Shaked M, et al (2006) Vimentin Binding to Phosphorylated Erk Sterically Hinders Enzymatic Dephosphorylation of the Kinase. J Mol Biol 364 938–944: 10.1016/j.jmb.2006.09.056 17046786

[77] Huelsenbeck SC, Rohrbeck A, Handreck A, Hellmich G, Kiaei E, et al (2012) C3 peptide promotes axonal regeneration and functional motor recovery after peripheral nerve injury. Neurotherapeutics 9: 85–98 10.1007/s13311-011-0072-y PMC3271155 21866396

[78] Boato F, Hendrix S, Hülsenbeck SC, Hofmann F, Groβe G, et al (2010) C3 Peptide enhances recovery from spinal cord injury by improved regenerative growth of descending fiber tracts. J Cell Sci 123: 1652–1662 10.1242/jcs.066050 20406886

[79] Auer M, Schweigreiter R, Hausott B, Thongrong S, Höltje M, et al (2012) Rho-independent stimulation of axon outgrowth and activation of the ERK and Akt signaling pathways by C3 transferase in sensory neurons. Front Cell Neurosci. 6: 43 10.3389/fncel.2012.00043 PMC3468917 23087613

[80] Auer M, Allodi I, Barham M, Udina E, Neiss WF, et al (2013) C3 exoenzyme lacks effects on peripheral axon regeneration in vivo. J Peripher Nerv Syst 18: 30–6 10.1111/jns5.12004 23521641

[81] Perlson E, Hanz S, Ben-Yaakov K, Segal-Ruder Y, Seger R, et al. (2005) Vimentin-dependent spatial translocation of an activated MAP kinase in injured nerve. Neuron 45: 715–26. DOI 10.1016/j.neuron.2005.01.023. 15748847

