# Supplementary figures and images for: Vimentin Mediates Uptake of C3 Exoenzyme
Source: PLoS One. 2014 Jun 26;9(6):e101071. doi: 10.1371/journal.pone.0101071 (PMC4072758; doi:10.1371/journal.pone.0101071)

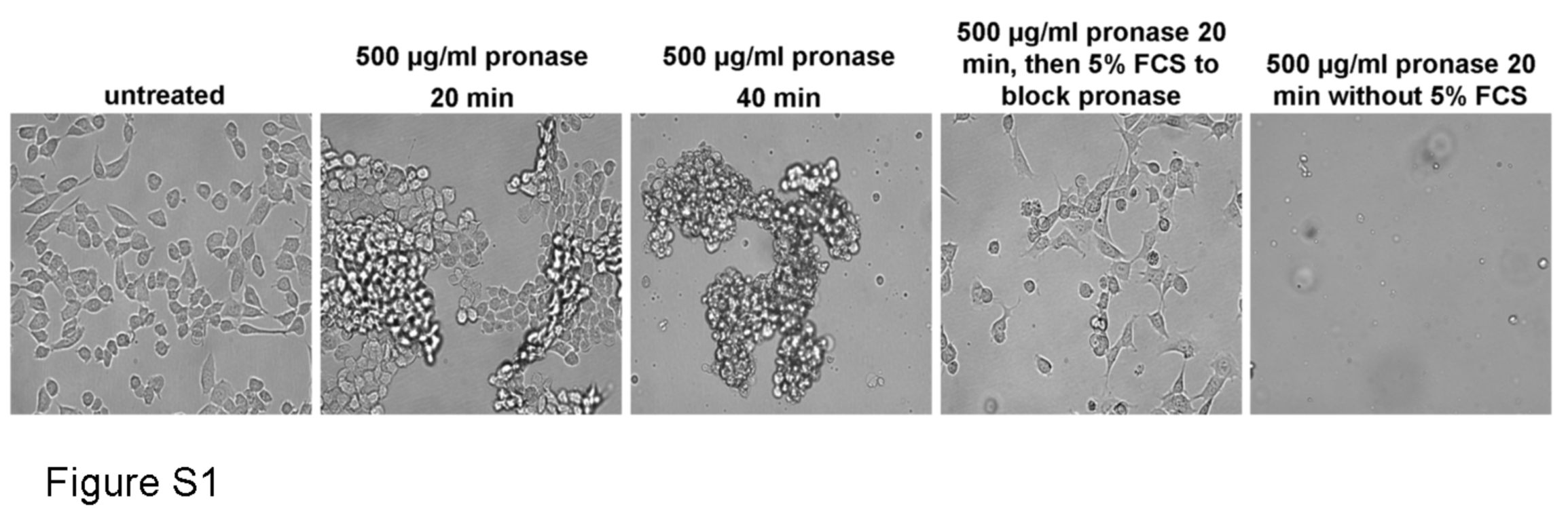

Supplement: Figure S1 — Pronase treatment resulted in cell detachment. HT22 cells were treated with 500 µg/ml of pronase for different incubation times at 4°C. The detached cells were analysed microscopically. Serum was added to detached cells to block pronase activity and cells were then washed with PBS and reseeded for 24 h at 37°C in media with 5% FCS. (TIF) [file pone.0101071.s001.tif]

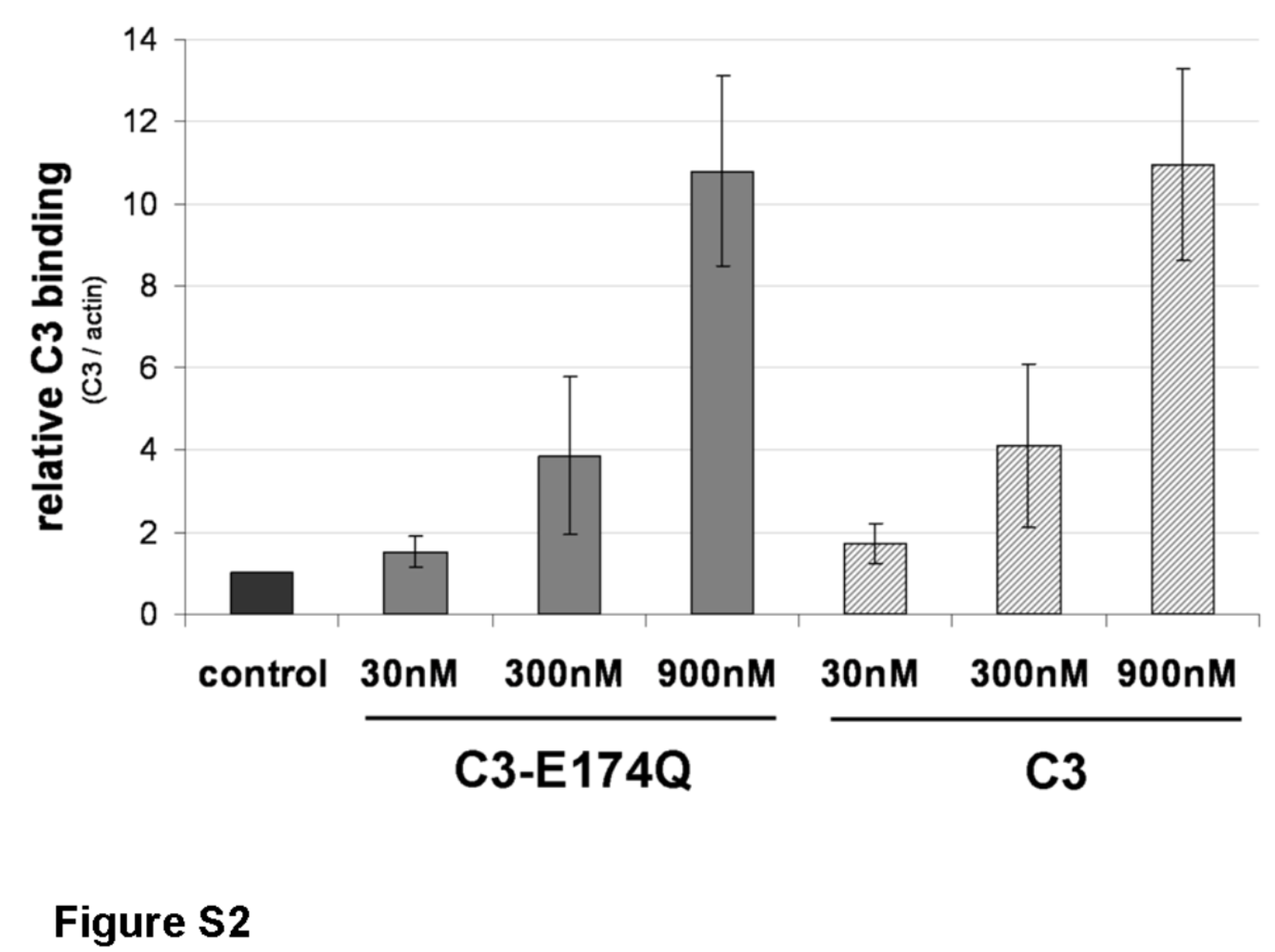

Supplement: Figure S2 — Binding of C3 and C3-E174Q to HT22 cells. HT22 cells were exposed to increasing concentrations of C3 ore C3-E174Q for 1 h at 4°C. Subsequently, C3bot and β-actin were detected by Western blot analysis. Densitometric quantification bound C3. All signal intensities of C3 were adjusted to the intensity of the corresponding β-actin signal. The differences in results were not statistically significant indicating that binding of C3 and C3-E174Q to HT22 cells is completing comparable. (TIF) [file pone.0101071.s002.tif]

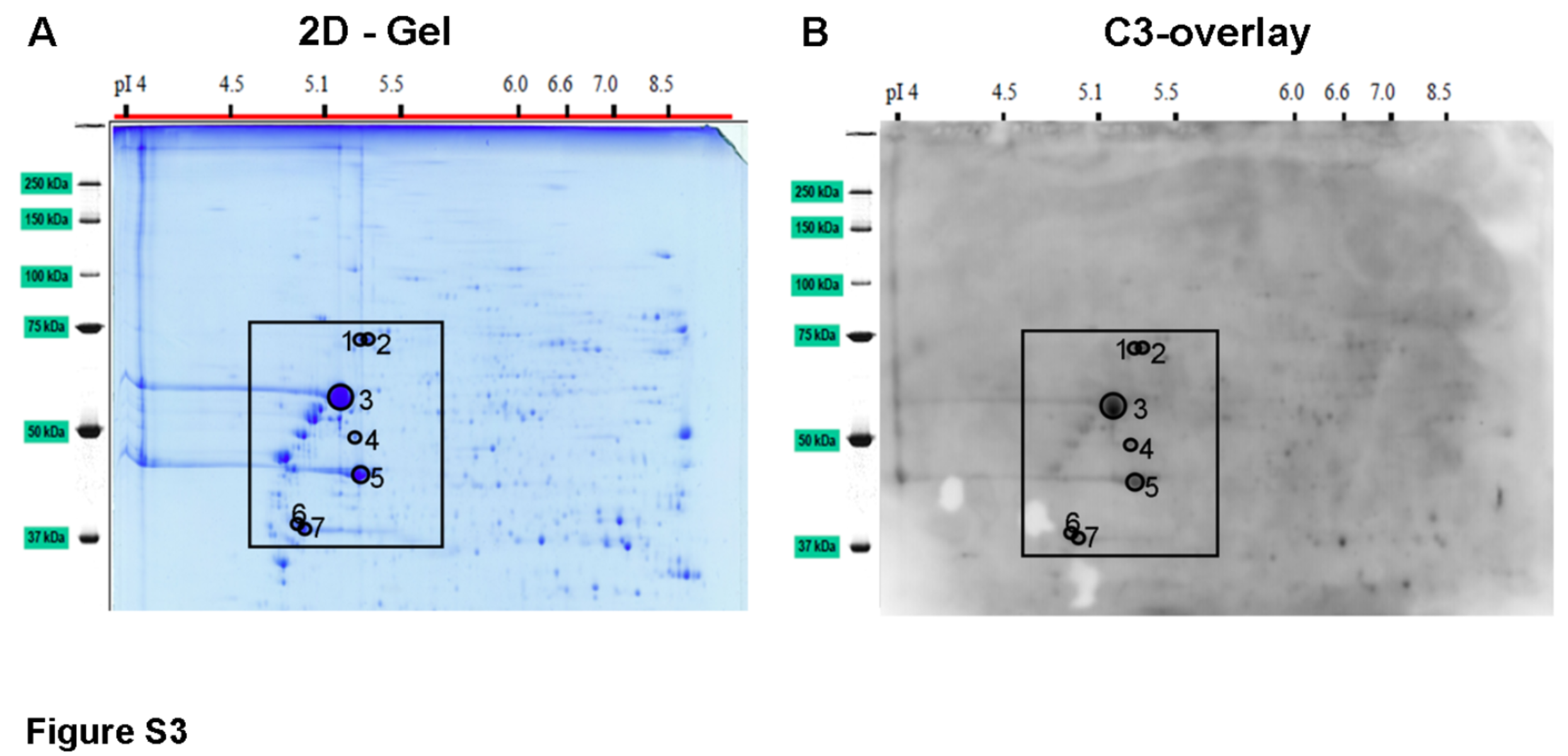

Supplement: Figure S3 — 2D-overlay experiments. Particulate fraction of HT22 cells were separated by 2D gel electrophoresis in the pH range 4–8, transferred to PVDF membrane and overlaid with C3 followed by immunoblotting and probing bound C3. Indicated positive spots in the 40–70 kDa region were in-gel digested with trypsin and subjected to mass spectrometry (spot 1 and 2 = HSP7C, spot 3 = vimentin, spot 4 = HNRPF, spot 5 = actin, spot 6 and 7 = nucleophosmin; Table S1, showing results for each spot). (TIF) [file pone.0101071.s003.tif]

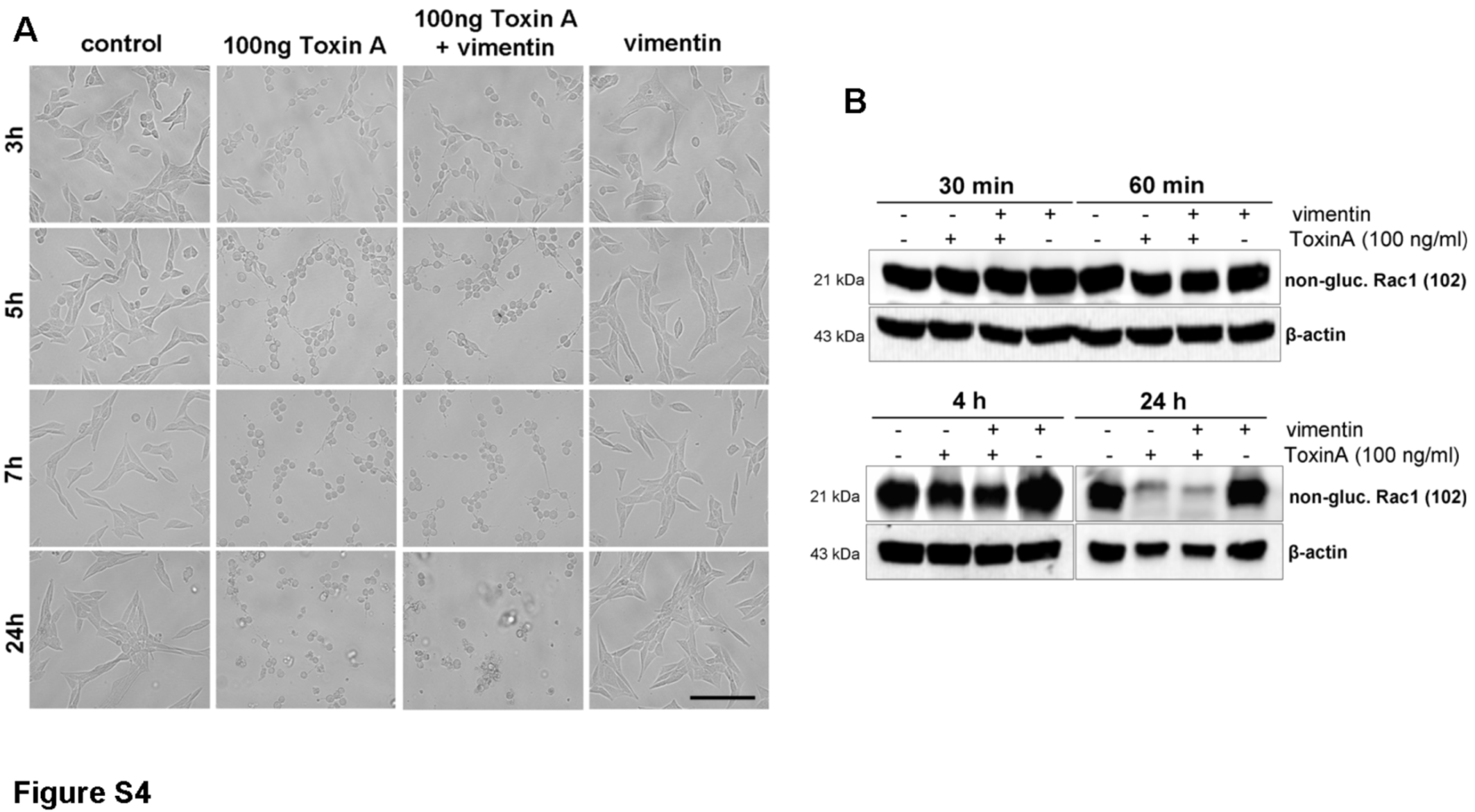

Supplement: Figure S4 — Effects in HT22 cells treated with Toxin A or Toxin A together with vimentin for the indicated times. A) Toxin A (100 ng/ml) induced morphological changes in HT22 cells within 3 h and this effect was more pronounced with longer incubation time. The combination of vimentin and Toxin A did not result in increase of morphological changes or faster cell rounding in comparison to Toxin A alone. Scale bar = 100 µM. B) The Rac glucosylation, which was assayed (after different time points as indicated) using a monoclonal anti-Rac1 antibody (Clone 102, BD Transduction Laboratories, Heidelberg, Germany), was in strong correlation with cell rounding (Figure S3A). (TIF) [file pone.0101071.s004.tif]

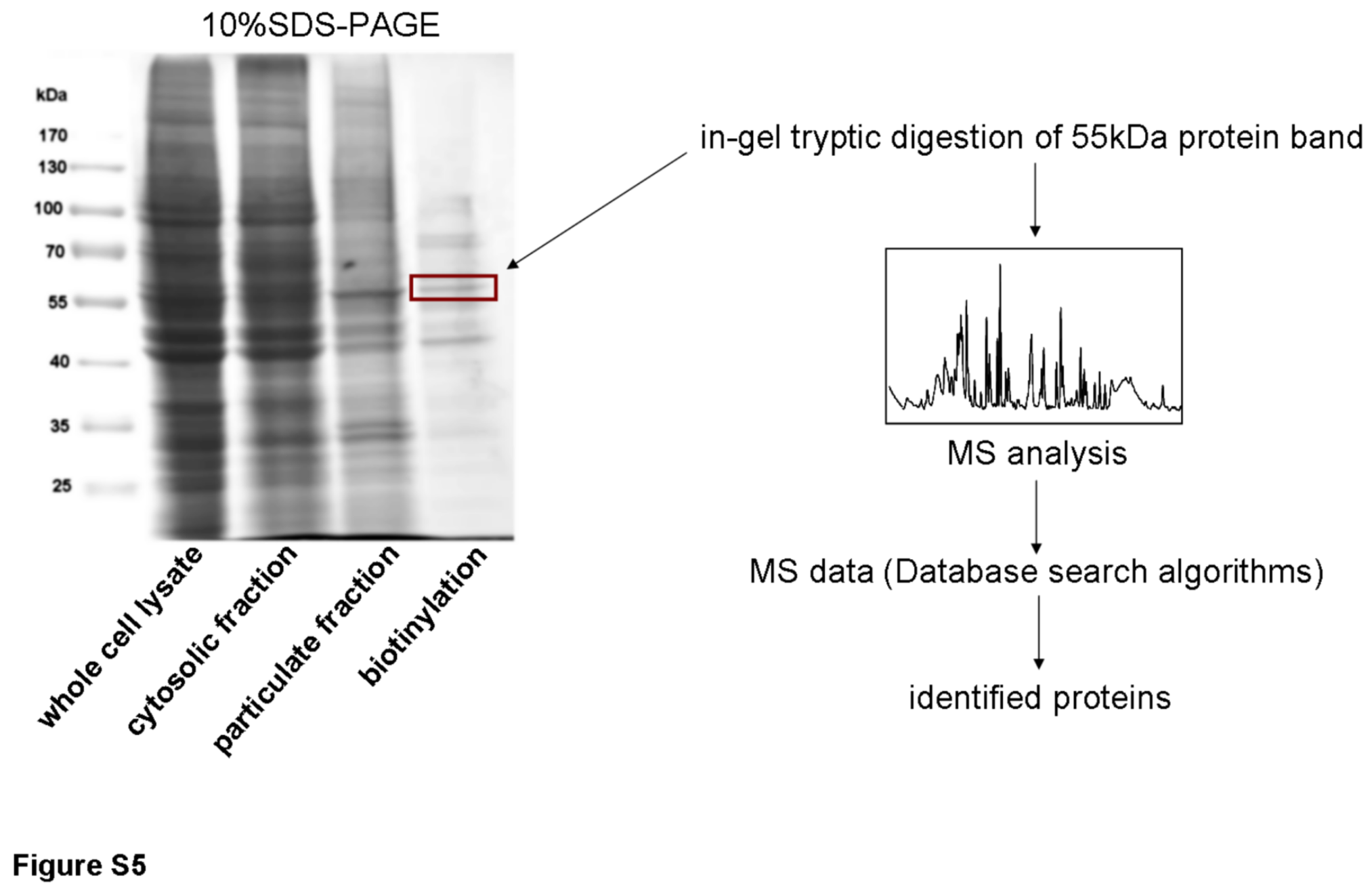

Supplement: Figure S5 — Schematic representation of protein identification with gel-based separation. Whole HT22 cell lysate, particulate fraction and biotin-labeled cell surface proteins were separated by 10% SDS-PAGE and stained with coomassie brilliant blue. The 55 kDa band of biotinylated sample was digested with trypsin and peptides were subjected to mass spectrometry analysis (Table S3, showing results of analysis). (TIF) [file pone.0101071.s005.tif]

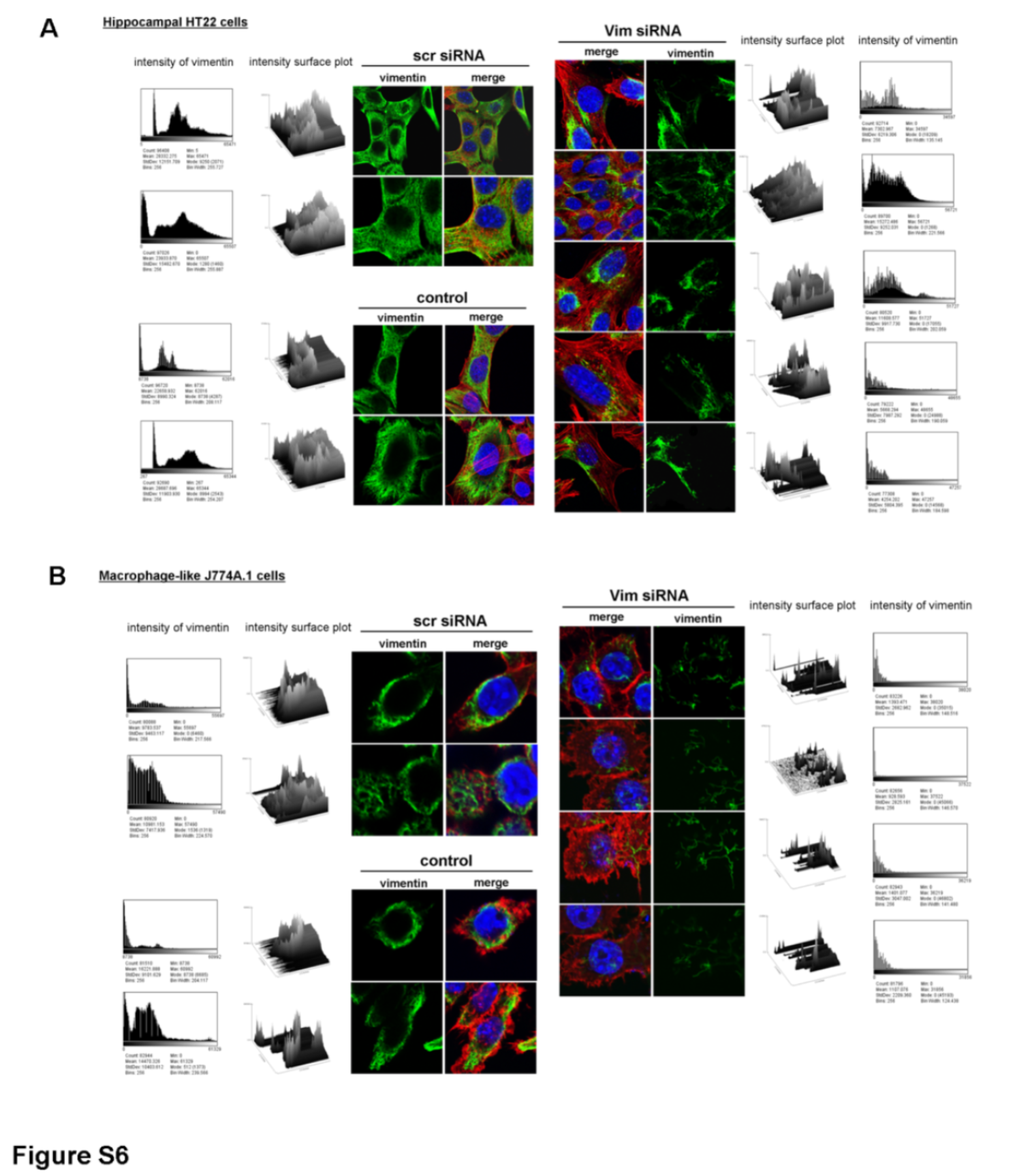

Supplement: Figure S6 — Detailed analysis of vimentin distribution after siRNA transfection. A) Results of hippocampal HT22 cells. In the middle of the panel were shown the immunhistochemical analysis as indicated. The quantification of vimentin intensity was done with ImageJ software. ImageJ creates a surface plot from all pixel intensities of vimentin to visualize the distribution of vimentin. Additionally, ImageJ calculates a grey level histogram of the image. In the histogram the x-axis represents the grey values and the y-axis shows the number of pixels. The histogram displays the distribution of grey values which correspondents to vimentin intensity (the lighter the gray value the higher the vimentin intensity). (TIF) [file pone.0101071.s006.tif]

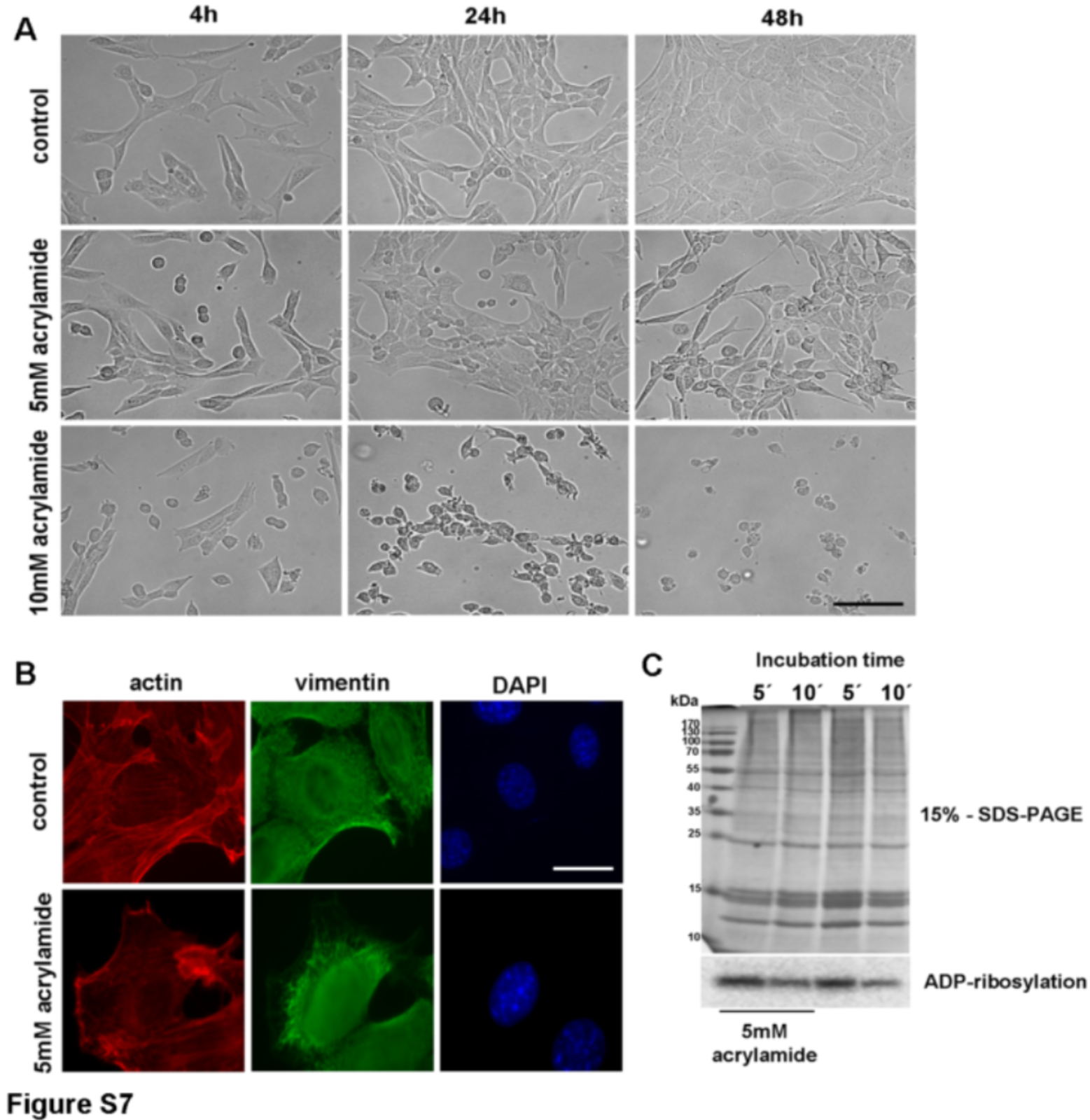

Supplement: Figure S7 — Effects of acrylamide on vimentin network and C3 enzyme activity. A) Morphological changes in HT22 cells exposed with 5 mM acrylamide for indicated times. Scale bar = 100 µM. B) Immunostaining with anti-vimentin mAb V9 and immunfluorescence microscopy was performed to study the distribution of vimentin filaments in acrylamide treated cells. The green anti-vimentin staining shows disruption in vimentin network in acrylamide treated cells. Scale bar = 20 µM. C) To rule out that acrylamide does not affect C3 enzyme activity ADP-ribosylation was performed in contemporary of acrylamide. Acrylamide pre-incubated HT22 cells were lysed. Subsequently, cell lysates were exposed to 1 µM C3 and 1 µCi [32P]NAD (Amersham Life Sciences, Arlington Heights, IL, USA) in 20 µl of 4× buffer containing 50 mM HEPES (pH 7.3), 10 mM MgCl2, 10 mM dithiothreitol, 10 mM thymidine and 10 µM NAD at 37°C for 5 or 10 min. The reaction was terminated by addition of Laemmli sample buffer, and then incubated at 95°C for 10 min. Samples were resolved by 15% SDS-PAGE, and the ADP-ribosylated Rho was analyzed by phosphorimaging (Cyclone, Packard American Instrument, MA, USA). (TIF) [file pone.0101071.s007.tif]

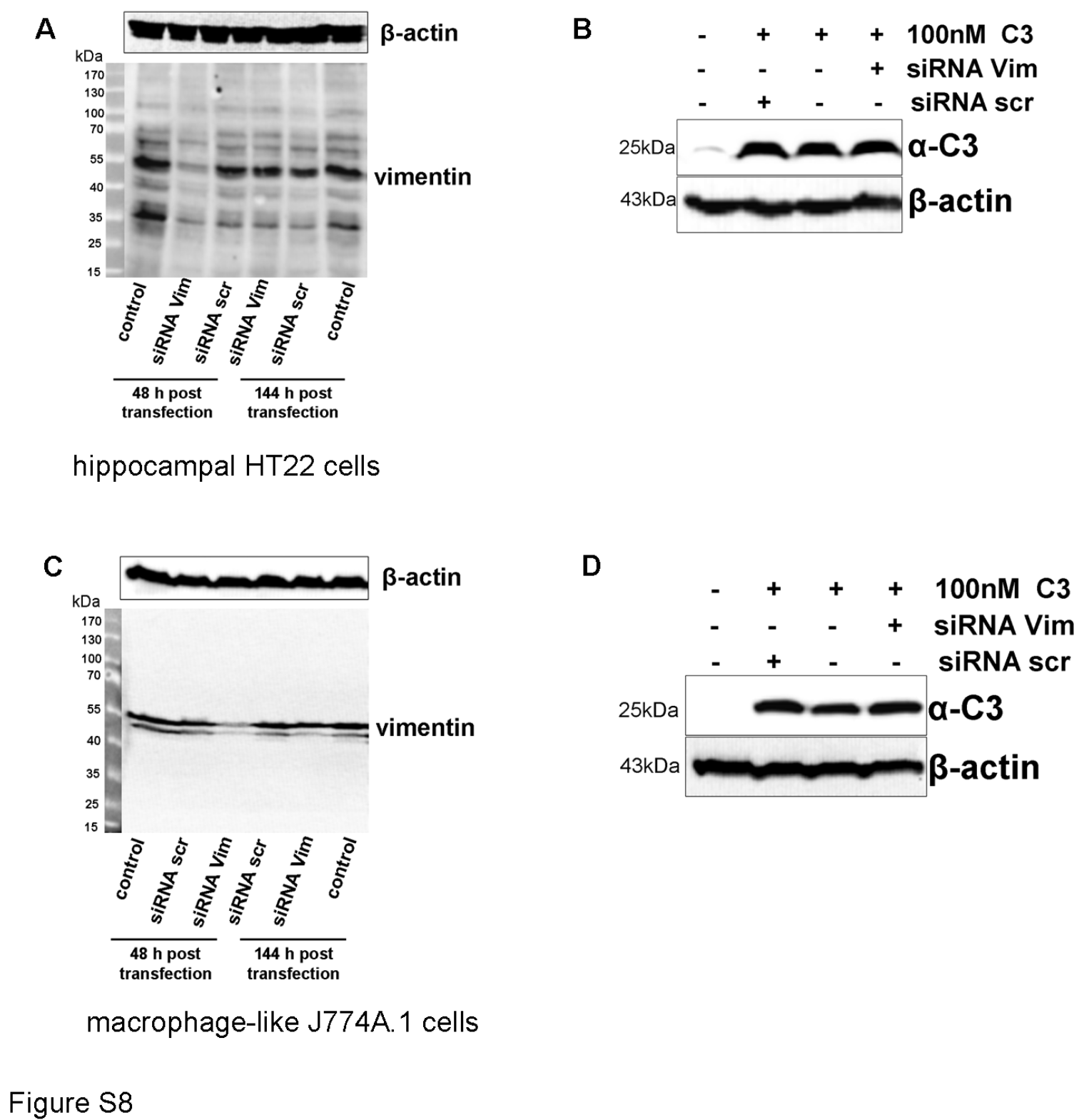

Supplement: Figure S8 — Binding of C3 to HT22 and J774A.1 cells 6 days after siRNA transfection. HT22 cells (A) and J774A.1 cells (C) were transfected with siRNA (scr = scrambled, Vim = vimentin). 144 h later Vimentin and β-actin were detected by Western blot analysis of cell lysates. 144 h after siRNA transfection, HT22 cells (B) and J774A.1 cells (D) were exposed to C3 (100 nM) for 1 h at 4°C. Bound C3 was detected in Western blot with anti-C3. β-actin was used as internal control. (TIF) [file pone.0101071.s008.tif]
