# Supplementary material for: Vimentin Mediates Uptake of C3 Exoenzyme
Source: PLoS One. 2014 Jun 26;9(6):e101071. doi: 10.1371/journal.pone.0101071 (PMC4072758; doi:10.1371/journal.pone.0101071)
Supplement: Table S1 — Identification of C3 interacting proteins by two-dimensional electrophoresis followed by C3-overlay and LC-MS/MS analysis. Results are LC-MS/MS data processed with Mascot search engine and the Swissprot database. (DOC) [file pone.0101071.s009.doc]

| Table S1 |  |  |  |  |
| --- | --- | --- | --- | --- |
| Suchparameter |  |  |  |  |
| Enzym | Trypsin |  |  |  |
| Peptide mass tol. | 20 ppm |  |  |  |
| Frag. Mass tol. | 0.7 Da |  |  |  |
| Swissprot | Mus musculus |  |  |  |
| Modifikation | Ox(M) |  |  |  |
|  | Carb(C) |  |  |  |
|  |  |  |  |  |
| Spot ID | Best Protein Accession | Best Protein Mass | Best Protein Score | Best Protein Description |
| Spot 1 | HSP7C_MOUSE | 71111 | 217 | Heat shock cognate 71 kDa protein OS=Mus musculus GN=Hspa8 PE=1 SV=1 |
| Spot 2 | HSP7C_MOUSE | 71111 | 327 | Heat shock cognate 71 kDa protein OS=Mus musculus GN=Hspa8 PE=1 SV=1 |
|  |  | 70053 | 138 | Heat shock-related 70 kDa protein 2 OS=Mus musculus GN=Hspa2 s GN=Dupd1 PE=2 SV=1 |
| Spot 3 | VIME_MOUSE | 53726 | 771 | Vimentin OS=Mus musculus GN=Vim PE=1 SV=3 |
| Spot 4 | HNRPF_MOUSE | 46127 | 288 | Heterogeneous nuclear ribonucleoprotein F OS=Mus musculus GN=Hnrnpf PE=1 SV=3 |
| Spot 5 | ACTB_MOUSE | 42136 | 412 | Actin, cytoplasmic 1 OS=Mus musculus GN=Actb PE=1 SV=1 |
|  |  | 42192 | 412 | Actin, cytoplasmic 2 OS=Mus musculus GN=Actg1 PE=1 SV=1 |
|  |  | 42418 | 241 | Actin, alpha cardiac muscle 1 OS=Mus musculus GN=Actc1 PE=1 SV=1 |
|  |  | 42450 | 241 | Actin, alpha skeletal muscle OS=Mus musculus GN=Acta1 PE=1 SV=1 |
|  |  | 42347 | 186 | Actin, gamma-enteric smooth muscle OS=Mus musculus GN=Actg2 PE=2 SV=1 |
|  |  | 42479 | 186 | Actin, aortic smooth muscle OS=Mus musculus GN=Acta2 PE=1 SV=1 |
|  |  | 42403 | 158 | Beta-actin-like protein 2 OS=Mus musculus GN=Actbl2 PE=1 SV=1 |
| Spot 6 | NPM_MOUSE | 32753 | 199 | Nucleophosmin OS=Mus musculus GN=Npm1 PE=1 SV=1 |
| Spot 7 | NPM_MOUSE | 32753 | 151 | Nucleophosmin OS=Mus musculus GN=Npm1 PE=1 SV=1 |

Table S1: Identification of C3 interacting proteins by two-dimensional electrophoresis followed by C3-overlay and LC-MS/MS analysis. Results are LC-MS/MS data processed with Mascot search engine and the Swissprot database.
