# Supplementary material for: Vimentin Mediates Uptake of C3 Exoenzyme
Source: PLoS One. 2014 Jun 26;9(6):e101071. doi: 10.1371/journal.pone.0101071 (PMC4072758; doi:10.1371/journal.pone.0101071)
Supplement: Table S2 — Complete list of proteins bound to C3, including accession No, Mascot detection score, number of identified peptides, mass, isoelectric point and sequence coverage. Results are LC-MS/MS data processed with Mascot search engine and the Swissprot database. (DOC) [file pone.0101071.s010.doc]

| Table S2 |  |  |  |  |  |  |  |
| --- | --- | --- | --- | --- | --- | --- | --- |
| Accession | Coverage | # Peptides | # AAs | MW [kDa] | calc. pI | Score | Description |
| P10853 | 23,81 | 5 | 126 | 13,9 | 10,32 | 143,50 | Histone H2B type 1-F/J/L OS=Mus musculus GN=Hist1h2bf PE=1 SV=2 - [H2B1F_MOUSE] |
| Q9CZX8 | 11,72 | 2 | 145 | 16,1 | 10,40 | 122,33 | 40S ribosomal protein S19 OS=Mus musculus GN=Rps19 PE=1 SV=3 - [RS19_MOUSE] |
| O88569 | 5,67 | 2 | 353 | 37,4 | 8,95 | 116,99 | Heterogeneous nuclear ribonucleoproteins A2/B1 OS=Mus musculus GN=Hnrnpa2b1 PE=1 SV=2 - [ROA2_MOUSE] |
| P60710 | 10,40 | 3 | 375 | 41,7 | 5,48 | 113,20 | Actin, cytoplasmic 1 OS=Mus musculus GN=Actb PE=1 SV=1 - [ACTB_MOUSE] |
| P20152 | 10,73 | 4 | 466 | 53,7 | 5,12 | 111,48 | Vimentin OS=Mus musculus GN=Vim PE=1 SV=3 - [VIME_MOUSE] |
| P15879 | 48,61 | 15 | 251 | 27,8 | 9,66 | 637,88 | Mono-ADP-ribosyltransferase C3 OS=Clostridium botulinum D phage GN=C3 PE=1 SV=2 - [ARC3_CBDP] |

Table S2: Complete list of proteins bound to C3, including accession No, Mascot detection score, number of identified peptides, mass, isoelectric point and sequence coverage. Results are LC-MS/MS data processed with Mascot search engine and the Swissprot database.
