# Supplementary material for: Vimentin Mediates Uptake of C3 Exoenzyme
Source: PLoS One. 2014 Jun 26;9(6):e101071. doi: 10.1371/journal.pone.0101071 (PMC4072758; doi:10.1371/journal.pone.0101071)
Supplement: Table S3 — Identified proteins in the biotinylated samples. Results are LC-MS/MS data processed with Mascot search engine and the Swissprot database. (DOC) [file pone.0101071.s011.doc]

| Table S3 |  |  |  |  |  |  |  |  |
| --- | --- | --- | --- | --- | --- | --- | --- | --- |
| Accession | Coverage | # PSMs | # Peptides | # AAs | MW [kDa] | calc. pI | Score | Description |
| P20152 | 77,90 | 82 | 48 | 466 | 53,7 | 5,12 | 2324,77 | Vimentin OS=Mus musculus GN=Vim PE=1 SV=3 - [VIME_MOUSE] |
| P27773 | 42,97 | 34 | 27 | 505 | 56,6 | 6,21 | 997,71 | Protein disulfide-isomerase A3 OS=Mus musculus GN=Pdia3 PE=1 SV=2 - [PDIA3_MOUSE] |
| P09103 | 41,65 | 32 | 27 | 509 | 57,1 | 4,91 | 962,52 | Protein disulfide-isomerase OS=Mus musculus GN=P4hb PE=1 SV=1 - [PDIA1_MOUSE] |
| O54724 | 40,05 | 23 | 16 | 392 | 43,9 | 5,52 | 944,88 | Polymerase I and transcript release factor OS=Mus musculus GN=Ptrf PE=1 SV=1 - [PTRF_MOUSE] |
| Q9D0K2 | 24,81 | 19 | 12 | 520 | 56,0 | 8,53 | 734,56 | Succinyl-CoA:3-ketoacid-coenzyme A transferase 1, mitochondrial OS=Mus musculus GN=Oxct1 PE=1 SV=1 - [SCOT1_MOUSE] |
| P09405 | 19,94 | 15 | 13 | 707 | 76,7 | 4,75 | 656,04 | Nucleolin OS=Mus musculus GN=Ncl PE=1 SV=2 - [NUCL_MOUSE] |
| P63038 | 29,49 | 18 | 18 | 573 | 60,9 | 6,18 | 651,17 | 60 kDa heat shock protein, mitochondrial OS=Mus musculus GN=Hspd1 PE=1 SV=1 - [CH60_MOUSE] |
| P80313 | 26,10 | 18 | 14 | 544 | 59,6 | 7,84 | 650,97 | T-complex protein 1 subunit eta OS=Mus musculus GN=Cct7 PE=1 SV=1 - [TCPH_MOUSE] |
| P80315 | 29,50 | 19 | 15 | 539 | 58,0 | 8,02 | 507,93 | T-complex protein 1 subunit delta OS=Mus musculus GN=Cct4 PE=1 SV=3 - [TCPD_MOUSE] |
| O08749 | 16,31 | 11 | 9 | 509 | 54,2 | 7,90 | 415,13 | Dihydrolipoyl dehydrogenase, mitochondrial OS=Mus musculus GN=Dld PE=1 SV=2 - [DLDH_MOUSE] |
| Q61753 | 19,70 | 10 | 10 | 533 | 56,5 | 6,54 | 401,16 | D-3-phosphoglycerate dehydrogenase OS=Mus musculus GN=Phgdh PE=1 SV=3 - [SERA_MOUSE] |
| P68373 | 20,94 | 11 | 8 | 449 | 49,9 | 5,10 | 389,65 | Tubulin alpha-1C chain OS=Mus musculus GN=Tuba1c PE=1 SV=1 - [TBA1C_MOUSE] |
| P14211 | 19,95 | 11 | 10 | 416 | 48,0 | 4,49 | 324,02 | Calreticulin OS=Mus musculus GN=Calr PE=1 SV=1 - [CALR_MOUSE] |
| P80314 | 11,78 | 9 | 7 | 535 | 57,4 | 6,40 | 314,93 | T-complex protein 1 subunit beta OS=Mus musculus GN=Cct2 PE=1 SV=4 - [TCPB_MOUSE] |
| P63017 | 17,34 | 8 | 8 | 646 | 70,8 | 5,52 | 296,79 | Heat shock cognate 71 kDa protein OS=Mus musculus GN=Hspa8 PE=1 SV=1 - [HSP7C_MOUSE] |
| Q91YH5 | 4,62 | 4 | 2 | 541 | 60,5 | 6,10 | 251,15 | Atlastin-3 OS=Mus musculus GN=Atl3 PE=2 SV=1 - [ATLA3_MOUSE] |
| Q8BXZ1 | 7,02 | 4 | 3 | 456 | 51,8 | 5,16 | 240,92 | Protein disulfide-isomerase TMX3 OS=Mus musculus GN=Tmx3 PE=1 SV=2 - [TMX3_MOUSE] |
| Q9CPY7 | 16,18 | 8 | 8 | 519 | 56,1 | 7,72 | 238,20 | Cytosol aminopeptidase OS=Mus musculus GN=Lap3 PE=1 SV=3 - [AMPL_MOUSE] |
| P38647 | 7,51 | 6 | 6 | 679 | 73,5 | 6,21 | 208,59 | Stress-70 protein, mitochondrial OS=Mus musculus GN=Hspa9 PE=1 SV=2 - [GRP75_MOUSE] |
| Q9CY58 | 14,99 | 4 | 4 | 407 | 44,7 | 8,54 | 184,58 | Plasminogen activator inhibitor 1 RNA-binding protein OS=Mus musculus GN=Serbp1 PE=1 SV=2 - [PAIRB_MOUSE] |
| P17225 | 9,30 | 7 | 7 | 527 | 56,4 | 8,34 | 173,06 | Polypyrimidine tract-binding protein 1 OS=Mus musculus GN=Ptbp1 PE=1 SV=2 - [PTBP1_MOUSE] |
| P24270 | 10,06 | 5 | 5 | 527 | 59,7 | 7,88 | 169,48 | Catalase OS=Mus musculus GN=Cat PE=1 SV=3 - [CATA_MOUSE] |
| P28656 | 11,76 | 4 | 3 | 391 | 45,3 | 4,46 | 155,09 | Nucleosome assembly protein 1-like 1 OS=Mus musculus GN=Nap1l1 PE=1 SV=2 - [NP1L1_MOUSE] |
| Q9D0F3 | 6,38 | 4 | 4 | 517 | 57,8 | 6,34 | 140,37 | Protein ERGIC-53 OS=Mus musculus GN=Lman1 PE=2 SV=1 - [LMAN1_MOUSE] |
| Q99MN9 | 8,13 | 4 | 4 | 541 | 58,4 | 7,47 | 131,00 | Propionyl-CoA carboxylase beta chain, mitochondrial OS=Mus musculus GN=Pccb PE=1 SV=1 - [PCCB_MOUSE] |
| P20029 | 5,50 | 5 | 4 | 655 | 72,4 | 5,16 | 129,98 | 78 kDa glucose-regulated protein OS=Mus musculus GN=Hspa5 PE=1 SV=3 - [GRP78_MOUSE] |
| P02535 | 3,33 | 2 | 2 | 570 | 57,7 | 5,11 | 122,31 | Keratin, type I cytoskeletal 10 OS=Mus musculus GN=Krt10 PE=1 SV=3 - [K1C10_MOUSE] |
| P99024 | 5,63 | 2 | 2 | 444 | 49,6 | 4,89 | 102,35 | Tubulin beta-5 chain OS=Mus musculus GN=Tubb5 PE=1 SV=1 - [TBB5_MOUSE] |
| P52293 | 4,35 | 2 | 2 | 529 | 57,9 | 5,68 | 102,07 | Importin subunit alpha-2 OS=Mus musculus GN=Kpna2 PE=1 SV=2 - [IMA2_MOUSE] |
| P62814 | 5,09 | 3 | 3 | 511 | 56,5 | 5,81 | 101,65 | V-type proton ATPase subunit B, brain isoform OS=Mus musculus GN=Atp6v1b2 PE=1 SV=1 - [VATB2_MOUSE] |

Table S3: Identified proteins in the biotinylated samples. Results are LC-MS/MS data processed with Mascot search engine and the Swissprot database.
