# Supplementary material for: Vimentin Mediates Uptake of C3 Exoenzyme
Source: PLoS One. 2014 Jun 26;9(6):e101071. doi: 10.1371/journal.pone.0101071 (PMC4072758; doi:10.1371/journal.pone.0101071)
Supplement: Table S4 — Proteins which interact with vimentin. n.d. = not described. (DOC) [file pone.0101071.s012.doc]

| Vimentin interacting protein | Vimentin domain | Reference |
| --- | --- | --- |
| *cross linker / membrane proteins* | | |
| polycystin-1 | rod domain | Xu et al., 2001 |
| plectin | rod and tail domain  rod domain | Sevcik et al., 2004  Favre et al., 2011 |
| desmoplakin | rod and tail domain | Lapouge et al., 2006 |
| kinesin | n.d. | Prahlad et al., 1998 |
| α2β1 integrin | n.d. | Kreis et al., 2005 |
| SNAP23 | n.d. | Faigle et al., 2000 |
| adapter complex AP-3 | n.d. | Styers et al., 2004 |
| filamin A | n.d. | Kim et al., 2010 |
| Hsp90 | n.d. | Zhang et al., 2006 |
| adenomatous polyposis coli (APC) | n.d. | Sakamoto et al., 2013 |
| formimidoyltransferase cyclodeaminase (FTCD) | n.d. | Gao et al., 2001 |
| *signal transduction* | | |
| 14-3-3 | head domain | Satoh et al., 2004 |
| pErk | rod domain | Perlson et al., 2006 |
| p38 MAPK | n.d. | Toda et al., 2012 |
| *toxins* | | |
| Pasteurella multocida toxin | head domain | Shime et al., 2002 |

TableS4: Proteins which interact with vimentin. n.d. = not described
